# Impaired NLRP3 inflammasome activation by chitin underlies refractory chromoblastomycosis caused by *Fonsecaea pedrosoi* muriform cells

**DOI:** 10.3389/fimmu.2026.1843553

**Published:** 2026-07-14

**Authors:** Yao Chen, Zilu Qu, Zhaolan Xie, Xiaowen Wang, Zhongsheng Tong, Luoyao Yang, Xu Zhang, Liuqing Chen, Bilin Dong

**Affiliations:** 1 Hubei Province & Key Laboratory of Skin Infection and Immunity, Department of Dermatology, Wuhan No.1 Hospital, Wuhan, Hubei, China; 2 Hubei Provincial Clinical Research Center for Infectious Skin Diseases, Department of Dermatology, Wuhan No.1 Hospital, Wuhan, Hubei, China; 3 Central Laboratory, Wuhan Wuchang Hospital, Wuhan, Hubei, China; 4 Research Center for Medical Mycology, Department of Dermatology and Venerology, Peking University First Hospital, Beijing, China

**Keywords:** bone-marrow-derived macrophages, chitin, chromoblastomycosis, *Fonsecaea pedrosoi*, muriform cells, NLRP3 inflammasome

## Abstract

**Introduction:**

Macrophage NLRP3 activation is crucial for antifungal immunity, yet its role in chromoblastomycosis—a chronic subcutaneous mycosis typically caused by *Fonsecaea pedrosoi*—remains unclear. Unlike prior self-resolving models using conidia and hyphae, clinical disease features muriform cells, a parasitic form resistant to macrophages and linked to chronicity. Notably, chitin, a weak NLRP3 agonist, accumulates on the surface of muriform cells. This study investigates whether chitin accumulation modulates NLRP3 activation and promotes fungal persistence.

**Methods:**

Human chromoblastomycosis lesions were analyzed by IHC (MPO, CD86, NLRP3) and RT-PCR for cytokine transcripts. WT and NLRP3–/– mice were footpad-inoculated with *F. pedrosoi* muriform cells for *in vivo* assessment. Flow cytometric bead array quantified IL-1β, IL-6, IL-10, TNF-α, and IL-17A in footpad homogenates, and the same panel (excluding IL-17A) in stimulated BMDM supernatants. NLRP3 pathway activation in BMDMs was confirmed by western blotting and immunofluorescence.

**Results:**

In human lesions, marked MPO+ neutrophil recruitment encapsulated muriform cells, yet multinucleated giant cells sequestered some fungal elements from neutrophils. Despite robust NLRP3 expression and elevated IL-1β, IL-6, TNF-α, and IL-10 versus normal skin (all p < 0.01), muriform cells persisted in tissue. In WT mice, lesions transitioned from purulent (day 7) to granulomatous (day 90), concurrent with declining IL-1β, IL-17A, IL-6, and TNF-α. MPO+ neutrophils abundantly surrounded muriform cells at day 7; at day 90, F4/80+ macrophages enveloped most fungi without elimination, forming multinucleated giant cells—recapitulating human pathology. In NLRP3–/– mice, IL-1β and IL-17A were produced in an NLRP3-independent manner and showed no decline over time. Muriform cells progressively budded and transitioned to hyphae both inside and outside giant cells; MPO+ neutrophils preferentially localized to hyphal areas, while macrophages remained around muriform cells, suggesting compartmentalized inflammation. *In vitro*, chitinase-treated muriform cells induced higher NLRP3-dependent IL-1β, IL-6, TNF-α, and IL-10 in WT BMDMs versus intact cells (all p < 0.01). Mechanistically, chitin accumulation partially suppressed NF-κB phosphorylation, reducing NLRP3 expression, caspase-1 cleavage, and ASC speck formation.

**Conclusion:**

NLRP3 mediates host defense against muriform cells yet fails to resolve chronic infection. Chitin accumulation on muriform cells attenuates NLRP3 activation, thereby promoting chromoblastomycosis chronicity.

## 1 Introduction

Chromoblastomycosis (CBM) is a subcutaneous mycosis caused by dematiaceous fungi in the order Chaetothyriales, which are usually introduced through traumatic inoculation (1). *Fonsecaea pedrosoi* and other related species in the genus *Fonsecaea* are recognized as the primary causative agents of chromoblastomycosis, especially in China (2, 3). The disease develops slowly but progressively, characterized by worsening verrucous hyperplasia with complications including lymphedema and mutilating damage to the extremities (4, 5). Recently, there have been a growing number of reports documenting the malignant transformation of persistent CBM lesions into squamous cell carcinoma (6–9). Thus far, the effects of antifungal therapies for this disease have remained inadequate (1, 4, 5). While CBM agents typically present as conidia or saprophytic hyphal form on media like potato dextrose agar (PDA) or Sabouraud dextrose agar (SDA), they exhibit a highly consistent morphology within human infected lesions, characterized by dark brown, swollen, thick-walled fungal cells with transverse septa-the muriform cells (also known as sclerotic bodies or Medlar bodies) (1, 5). Researches indicate that parasitic muriform cells possess a significantly greater capacity to withstand phagocytosis and evade subsequent immune clearance by host macrophages compared to their saprophytic conidia and hyphal counterparts (10, 11). The histological finding of muriform cells within infected tissues, particularly inside multinucleated giant cells (formed by macrophage fusion), is a key diagnostic criterion for CBM and a hallmark of its chronic progression (1, 5, 12). Therefore, understanding how muriform cells evade macrophage-mediated clearance is crucial to elucidate the chronicity of CBM.

Upon recognition of pathogen-associated molecular patterns (PAMPs) on fungal cell wall like β-glucan or α-mannan through their respective pattern recognition receptors (PRRs), human or murine macrophages initiate the intracellular “NOD-like receptor protein 3 (NLRP3)-ASC-Caspase-1” inflammatory signaling pathway. Its activation leads to the proteolytic processing (maturation) of pro-interleukin (IL)-1β and pro-IL-18 and their subsequent secretion (13–16). Together with tumor necrosis factor (TNF)-α and IL-6, these M1 macrophage-derived cytokines contribute to an inflammatory microenvironment that plays a pivotal role in host defense against fungal infections (10, 17). In contrast to β-glucan and mannan, chitin, a major skeletal component of the fungal cell wall, exhibits a markedly attenuated capacity to activate the NLRP3 inflammasome in both human and murine macrophages (15, 18–20). Notably, we have previously demonstrated that chitin is specifically accumulated on the surface of *F. pedrosoi* muriform cells during the morphological shift from saprophytic mycelia to this parasitic form (21, 22). Accordingly, we hypothesize that the muriform cells, through a “chitin accumulation effect” on their outer cell wall, actively inhibit the activation of the NLRP3 inflammasome and the secretion of M1 macrophage cytokines, thereby contributing to CBM refractoriness.

In this present study, we examined the infiltration of macrophages and neutrophils, as well as the expression pattern of NLRP3 and associated inflammatory factors within CBM-infected tissues. Furthermore, with an aim to investigate the role of NLRP3 signaling pathway in modulating host’s defense against the parasitic form of CBM agent, we established experimental CBM models by subcutaneously inoculating the *F. pedrosoi* muriform cells into the footpads of wild type (WT) and NLRP3-deficient (NLRP3–/–) mice, and compared the outcomes of infection between them. Additionally, chitinase digestion assay was performed to determine whether chitin accumulation on muriform cells would impair NLRP3 inflammasome activation and subsequent production of pro-inflammatory cytokines in murine bone marrow-derived macrophages (mBMDMs). Collectively, we hope to provide insights into the mechanisms of immune evasion in CBM.

## 2 Materials and methods

### 2.1 Sample collection

A total of six skin lesion biopsy samples were obtained from outpatients diagnosed with CBM according to histological identification of muriform cells at the Department of Dermatology, Wuhan No.1 Hospital (**Supplementary Figures 1A–F**), with corresponding formalin-fixed paraffin-embedded (FFPE) tissue blocks retrieved from the dermatopathology archives for three of these cases (**Supplementary Figures 1A–C**). Additionally, six samples of normal skin adjacent to either benign nevi or epidermal cysts were also included as controls. All samples were anonymized/coded in accordance with the Declaration of Helsinki. Written informed consent was obtained from each patient under protocols approved by the Institutional Review Board of Wuhan No. 1 Hospital (Project permit number: Kelun [2024] No.58).

### 2.2 Immunohistochemical staining for human MPO, CD68 and NLRP3

For human MPO, CD68 and NLRP3 IHC staining, after baking at 67 °C for 3 hours, sections (4 μm) prepared from FFPE tissue blocks were deparaffinized with Van-clear reagent for 60 min, and rehydrated through a graded ethanol series (100%, 95%, 80%, and 70%) to distilled water. Afterwards, heat-induced epitope retrieval was performed by immersing slides in citrate buffer (pH 6.0) and microwaving at medium-high power for a total of 15 min, ensuring the buffer remained just below boiling point. After washing with phosphate-buffered saline (PBS) three times (5 min, each), the sections were then incubated with 3% H_2_O_2_ for 20 min to quench endogenous peroxidase, and with 20% normal donkey serum (for MPO) or goat serum (for CD68 and NLRP3) for 20 min to block non-specific binding. Subsequently, sections were incubated overnight at 4°C with a goat anti-h/mMPO antibody (1:80, #AF3667, R&D systems), with a rabbit anti-hCD68 antibody (1:500, #76437, Cell Signaling) or with a rat anti-h/mNLRP3 antibody (1:50, #MAB7578, R&D systems). After washing with PBS as described above, the sections were incubated with an HRP-conjugated donkey anti-goat polyclonal antibody (1:1000 #GB23404, Servicebio), an HRP-conjugated goat anti-rabbit polymer detection system (ready-to-use, #G1312, Servicebio, S-vision) and an HRP-conjugated goat anti-rat polyclonal antibody (diluted 1:300, #GB23302, Servicebio) for 45 min at 37 °C, respectively. Finally, immunoreaction was visualized using 3, 3’-diaminobenzidine (DAB) (Lot: 2505F031; Servicebio). The development process was monitored microscopically to determine the optimal staining time, after which the reaction was terminated with distilled water. Counterstaining was carried out with hematoxylin. Stained sections were imaged using a Nikon Eclipse Ts2R microscope fitted with a Nikon Digital Sight 10 microscope camera. Positive staining was characterized as follows: brown granular deposits localized to the cytoplasm for MPO or CD68, cytoplasmic or nuclear positivity for NLRP3. All sections were counterstained with hematoxylin to visualize nuclei.

### 2.3 RT-qPCR analysis of cytokine mRNA expression in CBM tissues

For RT-qPCR assay, six CBM specimens and six control samples of normal skin adjacent to either benign nevi or epidermal cysts were collected. Briefly, tissues were homogenized in TRIzol reagent (Invitrogen, China). The RT reactions were carried out using a SYBR green-containing PCR kit (Qingke, China). Primer sequences are listed in **Table 1**. GAPDH acted as an internal reference to the gene. Gene expression levels were quantified using the 2^⁻ΔΔCt^ method. All RT-PCR assays were performed using an ABI Stepone Plus Real-Time PCR System (Applied Biosystems, USA).

**Table 1:** Primer sequences for human (h) or mouse (m) RT-PCR.

Gene	Forward primer (5’-3’)	Reverse primer (5’-3’)	Tm (°C)
hIL-1β	CCAAACCTCTTCGAGGCACA	GCTGCTTCAGACACTTGAGC	60
hIL-6	TTCGGTCCAGTTGCCTTCTC	CAGCTCTGGCTTGTTCCTCA	60
hTNF-α	GACAAGCCTGTAGCCCATGT	GGAGGTTGACCTTGGTCTGG	60
hIL-10	TGCAAAAGAAGGCATGCACAG	GTTCACATGCGCCTTGATGT	60
hGAPDH	TCGGAGTCAACGGATTTGGT	AGTGATGGCATGGACTGTGG	60
mIL-1β	AGAGCCCATCCTCTGTGACT	CTCTGCTTGTGAGGTGCTGA	60
mIL-18	AGAAAGCCGCCTCAAACCTT	TCAGTCTGGTCTGGGGTTCA	60
mGAPDH	TCAGGAGAGTGTTTCCTCGT	CCTTGACTGTGCCGTTGAAT	60

### 2.4 Fungal strain and *in vitro* induction of muriform cells

The *F. pedrosoi* strain WH10–002 was isolated from the skin lesion of a CBM patient and identified via sequencing of the internal transcribed spacer (ITS) region NCBI GenBank accession no. GQ420654.1 (Linkage: https://www.ncbi.nlm.nih.gov/nuccore/GQ420654.1). The strain was cultivated on potato dextrose agar (PDA) supplemented with chloramphenicol (50 μg/mL) at 28 °C and periodically transferred at 60-day intervals for preservation.

Prior to further experiments, the stock culture was subjected to slide culture on Potato dextrose agar (PDA) at 28 °C for 7–10 days and morphologically confirmed as a pure culture of the genus *Fonsecaea* (**Supplementary Figure 2A**).

To induce the formation of muriform cells, the stock culture was inoculated into the Sabouraud Dextrose Broth (SDB) (DifcoTM, BD), and cultured for 2 weeks at 28°C for hyphal preparation. Then, the mycelia grown in SDB were unfolded with a glass homogenizer, filtered through a nylon filter (200 mesh), and adjusted to a final concentration of 0.5×10^6^/mL separated short hyphal fragments. 500 μL of hyphal fragments was re-inoculated into 30 mL of synthetic basal medium (ATCC medium 830), pH 5.5, with the following composition (g/L): MgSO4, 0.1; NH_4_NO_3_, 1.5; KH_2_PO_4_, 1.8; Biotin, 5×10^-5^; thiamine-HCl, 1.0×10^-4^; Glycerol, 6.5, as previously described (5). In addition, Nikkomycin Z (N8028, Sigma-aldrich) was added at a final concentration of 50 μg/mL (5). During the 60-day incubation period at 36°C, the formation of muriform cells was confirmed by microscopic examination (Nikon Eclipse Ts2R). For functional experiments both *in vivo* and *in vitro*, the induced muriform cells were gently dispersed and filtered as described to eliminate residual hyphal fragments. Subsequently, the purity of muriform cells in the filtrate was determined by calculating their mean percentage relative to the total cell count across at least 10 randomly selected microscopic fields (**Supplementary Figures 2B, C**).

### 2.5 Mice

All animal experiments in this study were performed in accordance with the recommendations in the Guide for the Care and Use of Laboratory Animals of National Institutes of Health. Our study protocol was approved by the Institutional Animal Care and Use Committee of Wuhan No.1 Hospital (project license number: WHB202203013). To minimize suffering, mice were anaesthetized prior to inoculation or sacrifice.

C57BL/6n NLRP3–/– mice (SN: KOCMP-216799-Nlrp3-B6N-VA) (n=6; 2 males and 4 females; 8–10 weeks old) were purchased from Cyagen Suzhou Biosciences Technology Co., Ltd. (Suzhou, China), and subsequently bred and maintained in special pathogen-free (SPF) conditions. Male C57BL/6n WT mice (SPF, n = 32, 5 weeks old) were purchased from Beijing Vital River Laboratory Animal Technology Co., Ltd. (Beijing, China) and allowed to acclimate for one week prior to use.

### 2.6 Footpad infection with *F. pedrosoi*-muriform cells

The muriform cell aggregates in ATCC 830 medium were unfolded and filtered as described in section 2.4. The filtrate containing dispersed muriform cells was further washed twice in 0.9% saline by centrifugation at 4000 rpm for 8 min, and finally adjusted to a concentration of 1.5×10^8^ cells/mL for use. The viability rate of muriform cells was detected by FUN1 cell stain (Cat: F7030, Invitrogen™) using flow cytometer (CytoFLEX S; Beckman Coulter, Inc., CA, USA) according to the protocol and reached above 96%.

Both the WT and NLRP3–/– groups of mice (6 weeks old) were used in this study. Prior to inoculation, the mice were anaesthetized by intraperitoneal injection with 0.4 μL of Anasedan and 0.35 ml of Dopalen per kg body weight. Then, 100 μL of prepared muriform cell suspension (1.5×10^8^ cells/mL) was injected subcutaneously into each rear footpad (n = 27 per group). The mice injected subcutaneously with 100 μL of saline were set as the vehicle controls (n = 3 per group).

### 2.7 Evaluation of footpad lesion and fungal burden

The development of footpad lesion for the same three mice randomly selected from each infected group was monitored twice a week for up to a maximum of 90 days, and footpad swelling was simultaneously measured using a vernier caliper. Footpad volume was calculated by length×width×thickness measurements, and the data were represented as mean ± standard deviation (SD) for the infected groups as well as the vehicle controls. Images of the same infected footpad were taken consecutively on day 0 (pre-inoculation) and at 7, 15, 21, 28, 35, 45, 60, 75, and 90 days post-inoculation (dpi).

For fungal burden counting, both groups of infected mice were randomly sacrificed at 7, 15, 28, 45, 60 and 90 dpi (n = 4 per group per time point). One infected rear footpad from each mouse was aseptically excised, minced and homogenized in sterile saline using glass homogenizers. The homogenates were then filtered through a 200-mesh nylon sieve, and 2 mL of filtrate was serially diluted 10-fold (from 1 to 10^4^) in saline supplemented with an antibiotic cocktail containing 1000 U/mL penicillin, 1000 μg/mL streptomycin, 100 μg/mL gentamicin, and 50 μg/mL chloramphenicol. Aliquots (100 μL) of each dilution were plated in triplicate on PDA plates supplemented with 50 μg/mL chloramphenicol and incubated at 28 °C for 5 days. Fungal burden was quantified by counting colony-forming units (CFU) at suitable dilutions and expressed as CFU per footpad.

### 2.8 H&E and IHC staining of infected footpads

For hematoxylin and eosin (H&E) staining, skin biopsy samples were obtained from the contralateral infected rear footpads of the same mice used for fungal burden quantification at 7, 45, and 90 dpi, respectively. The specimens were fixed in 4% neutral buffered paraformaldehyde for 72 h, and then embedded in paraffin for histological processing. Sections (4 μm thick) were prepared, deparaffinized, and stained with hematoxylin and eosin (H&E).

For mouse MPO, F4/80 and NLRP3 IHC staining, deparaffinized sections from both groups at 7 and 90 dpi were used. Briefly, the experimental procedures of antigen retrieval, endogenous peroxidase inactivation, and blocking with 20% donkey serum (for MPO) or 20% goat serum (for F4/80 and NLRP3) were performed sequentially, as detailed in section 2.2. Then, the sections were incubated overnight at 4°C with a goat anti-h/mMPO antibody (1: 80, #AF3667, R&D systems), with a rabbit anti-mF4/80 antibody (1: 300, #70076, Cell Signaling) or with a rat anti-h/mNLRP3 antibody (1: 50, #MAB7578, R&D systems). Subsequently, the reagent selection and operating protocols for secondary antibody incubation, DAB color development, and hematoxylin counterstaining were still performed according to the procedures described in section 2.2.

H&E-stained and IHC-stained sections were imaged using the microscope described in section 2.2. Positive staining was characterized as follows: brown granular deposits localized to the cytoplasm for MPO; cell membrane (and partially cytoplasmic) positivity for F4/80; and cytoplasmic or nuclear positivity for NLRP3. All sections were counterstained with hematoxylin to visualize nuclei.

### 2.9 Heat inactivation and chitinase treatment of *F. pedrosoi* muriform cells

To ensure morphological stability in subsequent cellular experiments, *in vitro*-induced *F. pedrosoi* muriform cells were heat-inactivated at 70 °C for 90 min. Then, chitinase treatment was performed according to the method described in our previous publication with minor modifications (21, 22). Specifically, a total of 1.0×10^8^ cells were incubated with 5 mL PBS containing 25 U chitinase (#C6137, Sigma-aldrich) at 37°C overnight with shaking at 160 rpm. Finally, the muriform cells were washed five times with PBS, centrifuged at 4, 000 rpm for 10 min per wash, and resuspended in 1 mL PBS for use.

### 2.10 FITC-conjugated WGA staining for chitin distribution

Wheat germ agglutinin (WGA) primarily binds to N-acetylglucosamine (GlcNAc), the main component of chitin. To verify chitin distribution on the surface of muriform cells, cells were stained with FITC-conjugated WGA (1 mg/mL; #L4895, Sigma-Aldrich) according to the manufacturer’s protocol and our previous studies (21, 22). Briefly, after culture in ATCC 830 medium for 35 d or 60 d, muriform cells were heat-inactivated, with or without chitinase treatment, and then adjusted to a final concentration of 2.0×10^6^ cells in 100 µL of PBS containing 2% BSA. Subsequently, these muriform cells were incubated with 10 μl of FITC-conjugated WGA at 4 °C for 90 min, washed three times with PBS, and then resuspended in 1 mL of ice-cold PBS. Finally, the binding of WGA on the surface of muriform cells was measured using confocal laser scanning microscopy (STELLARIS 5 WLL, Leica). Fluorescence was detected with excitation at 488 nm and emission at 510–540 nm.

### 2.11 Cytokines detection in the footpad lesion of WT and NLRP3–/– mice infected with *F. pedrosoi* muriform cells

Prior to cytokine measurement, cryo-preserved footpad tissues from WT and NLRP3−/− mice (infected with *F. pedrosoi* muriform cells at 7, 45, and 90 dpi) and from N.S.-inoculated controls were minced into small fragments in 2-mL microcentrifuge tubes. The samples were then supplemented with 600 µL of N.S. and a combination of 1-mm and 3-mm zirconia beads, and homogenized using a TissueLyser (QIAGEN, Hilden, Germany) at 30 Hz for two 5-min cycles, with ice-water bath cooling (5 min) applied before and between cycles to prevent thermal degradation. The resultant homogenates were centrifuged at 4, 000rpm for 5 min at 4 °C. The supernatants were transferred to new micro-centrifuge tubes and re-centrifuged under identical conditions to remove tissue debris. The final supernatants were then collected for further analysis.

The levels of IL-1β, IL-6, IL-10, IL-17A and TNF-α in the supernatants were quantified using the Mouse Cytometric Bead Array (CBA) Flex Set with Master Buffer Kit according to the manufacturer’s protocol (Capture mAbs: IL-1β, E5 #560232; IL-6, B4 #558301; IL-10, C4 #558300; IL-17A, C5, #560283; TNF-α, C8 #558299; Master Buffer Kit: #558266; BD Bioscience Pharmingen, SanDiego, CA, USA). In brief, target cytokines were captured using bead-immobilized specific capture antibodies, with the beads distinguished by their unique APC/APC-A750 fluorescence intensities (**Supplementary Figures 3A–C**). Subsequently, the target cytokines were detected via the addition of corresponding phycoerythrin (PE)-conjugated detection antibodies from the Flex Set using a CytoFLEX S flow cytometer (Beckman Coulter, Suzhou, China) (**Supplementary Figures 3D–F**). Cytokine concentrations were quantified using the PE mean fluorescence intensity (MFI), which was interpolated against a standard curve generated from serially diluted cytokine standards. This analysis was performed with FCAP Array V3.0 software (**Supplementary Figures 3A–G**). As a negative control, assay diluent was processed in parallel with the samples.

Notably, due to the limited availability of archived samples (n = 1 per time point at 7, 45, and 90 days post-infection), statistical testing was not applicable. Nevertheless, we provide the time-course data in the **Supplementary Materials** (**Supplementary Figures 3D–G**) to illustrate the overall trends.

### 2.12 mBMDMs culture, stimulation, and cytokines detection

mBMDMs were isolated from the femurs and tibiae of C57BL/6n WT and NLRP3–/– mice, and differentiated into macrophages for 7 days in Dulbecco’s Modified Eagle Medium (DMEM) supplemented with 10% fetal bovine serum (FBS) and 40 ng/mL macrophage colony-stimulating factor (M-CSF) under 5% CO_2_. To assess mBMDM maturation, we analyzed F4/80 expression by flow cytometry. Briefly, cells were stained with an FITC-conjugated Rat anti-mouse F/80 antibody (#123107, Biolegend) for 30 min at room temperature according to the manufacturer’s protocol, and analyzed on a CytoFLEX S flow cytometer (Beckman Coulter, Suzhou, China).

Subsequently, cells grown in 6-well plates (1.0×10^6^ cells/well) were stimulated for 14 h with heat-inactivated muriform cells (MOI = 3:1), with or without chitinase pretreatment. All stimulations were performed in triplicate, while untreated mBMDM wells served as controls. Culture supernatants from stimulated and control wells were harvested and centrifuged at 3000 rpm for 5 min to remove cellular debris.

The levels of IL-1β, IL-6, IL-10, and TNF-α in the supernatants were quantified using the Mouse Cytometric Bead Array (CBA) Flex Set with Master Buffer Kit as mentioned above (**Supplementary Figures 4A–K**). The capture beads responsible for these cytokines and the corresponding phycoerythrin (PE)-conjugated detection antibodies were the same as those in Section 2.11.

IL-18 levels in the supernatants were measured using a commercial enzyme-linked immunosorbent assay (ELISA) kit (biotinylated anti-mouse IL-18 antibody and HRP-labeled avidin; Cat# E-EL-M0730, Elabscience, China) according to the manufacturer’s instructions. The optical density was determined with a SpectraMax 190 absorbance microplate reader (Molecular Devices, Suzhou, Jiangsu, China), and cytokine concentrations were calculated against a standard curve.

Additionally, IL-1β and IL-18 mRNA levels in WT mBMDMs were measured before and after 14 h stimulation with heat-inactivated muriform cells (MOI = 3:1), with or without chitinase pretreatment. For RT-qPCR assay, the cells were harvested after wash with PBS, and homogenized in TRIzol reagent (Invitrogen, China). The RT reactions were carried out using a SYBR green-containing PCR kit (Qingke, China). Primer sequences are listed in **Table 1**. GAPDH acted as an internal reference to the gene. Gene expression levels were quantified using the 2^⁻ΔΔCt^ method. All RT-PCR assays were performed using an ABI Stepone Plus Real-Time PCR System (Applied Biosystems, USA).

### 2.13 Detection of NLRP3 inflammasome activation in mBMDMs

NLRP3 priming, indicated by NF-κB phosphorylation and increased NLRP3 expression, and subsequent Caspase-1 cleavage were determined by Western blotting. Briefly, mBMDMs were stimulated as described in section 2.11 and lysed in RIPA buffer containing a protease inhibitor cocktail (#BL612A, Biosharp, China). Protein concentration was determined using a BCA protein assay kit (#P0012S, Beyotime, China). Equal amounts of protein (20 μg) were separated by 10% SDS-PAGE and transferred onto PVDF membranes (Millipore, USA). After blocking with 5% non-fat milk, the membranes were incubated overnight at 4 °C with primary antibodies targeting mouse proteins. The antibodies used were as follows: rabbit anti-NF-κB monoclonal antibody (1:1000, #8242, Cell Signaling); rabbit anti-phospho-NF-κB (p65) monoclonal antibody (1:1000, #3033, Cell Signaling); rat anti-NLRP3 monoclonal antibody (1:250, #MAB7578, R&D Systems); rabbit anti-pro-caspase-1 (p45) monoclonal antibody (1:5000, #87271-4-RR, Proteintech); rabbit anti-cleaved-caspase-1 (p20) monoclonal antibody (1:1000, #4199, CST), and rabbit anti-β-actin monoclonal antibody (1:1000, #4970, Cell Signaling). Following washes with TBST five times (15 min per wash) on a shaker, membranes were incubated for 1h at room temperature with corresponding HRP-conjugated secondary antibodies, including goat anti-rat IgG (H+L) (1:5000, #GB23302, Servicebio) and goat anti-rabbit IgG (H+L) (1:2000, #SA00001-2, Proteintech). Signals were visualized using UltraSignal hypersensitive ECL chemiluminescence substrate (4A Biotech, China) on a ChemiDoc Imaging System (Bio-Rad, USA), and the densitometric analysis of the bands was performed using ImageJ software.

Additionally, ASC speck formation, a critical indicator of NLRP3 inflammasome activation, was assessed by immunofluorescence staining. Specifically, 2 mL mature mBMDMs were re-seeded into CLSM-specific dishes (35 mm, Corning, USA) at a density of 1× 10⁵, and stimulated as described in section 2.5 when the cells reached 80-90% confluence. The cells were fixed with 4% paraformaldehyde for 20 min, permeabilized with 0.1% Triton X-100 for 10 min, and then incubated with rabbit-derived anti-mouse ASC antibody (1:400, #67824, Cell Signaling) overnight at 4 °C. Subsequently, the cells were incubated with Alexa Fluor 594-conjugated goat-derived anti-rabbit antibody (1:1000, #8889, CST) for 1 h at room temperature. Nuclei were stained with DAPI. ASC speck formation in stimulated or untreated mBMDMs was analyzed, and images were acquired using a confocal microscope (STELLARIS 5 WLL, Leica). The excitation/emission wavelengths were set at 405 nm/425–460 nm for DAPI, and 594 nm/615–670 nm for Alexa Fluor 594, respectively. The criteria for ASC speck formation was defined as a single, dense, cytoplasmic punctate structure, representing the aggregation of ASC proteins upon inflammasome activation. To quantify the extent of speck formation, the percentage of cells that contained an ASC speck was counted in the whole section.

### 2.14 Statistical analysis

Statistical analyses were conducted using IBM SPSS Statistics 22.0 (IBM Corp., Armonk, NY, USA) and GraphPad Prism (version 10). Quantitative data were expressed as mean ± standard deviation (SD). Normality of the data was assessed using the Shapiro–Wilk test, and homogeneity of variances was verified using Levene’s test. All data met the normality assumption (p > 0.05) and the homogeneity of variances assumption (p > 0.05), justifying the use of parametric tests. Comparisons between two independent groups were made using the two-tailed Student’s t-test. Repeated-measures analysis of variance (ANOVA) was employed to analyze data collected at multiple time points. For comparisons among multiple groups, one-way ANOVA followed by *post hoc* LSD-t test was performed. A p value of < 0.05 was considered statistical significance.

Additionally, statistical comparisons were not applied to the cytokine time-course data from footpad tissues due to the limited sample availability (n=1 per time point at 7, 45, and 90 days post-inoculation). These data are shown descriptively in the **Supplementary Materials** (**Supplementary Figure 3**).

## 3 Results

### 3.1 Defective human macrophage killing and multinucleated giant cell formation sequester muriform cells from neutrophils, with robust NLRP3 expression and cytokine heterogeneity

**Figure 1:**
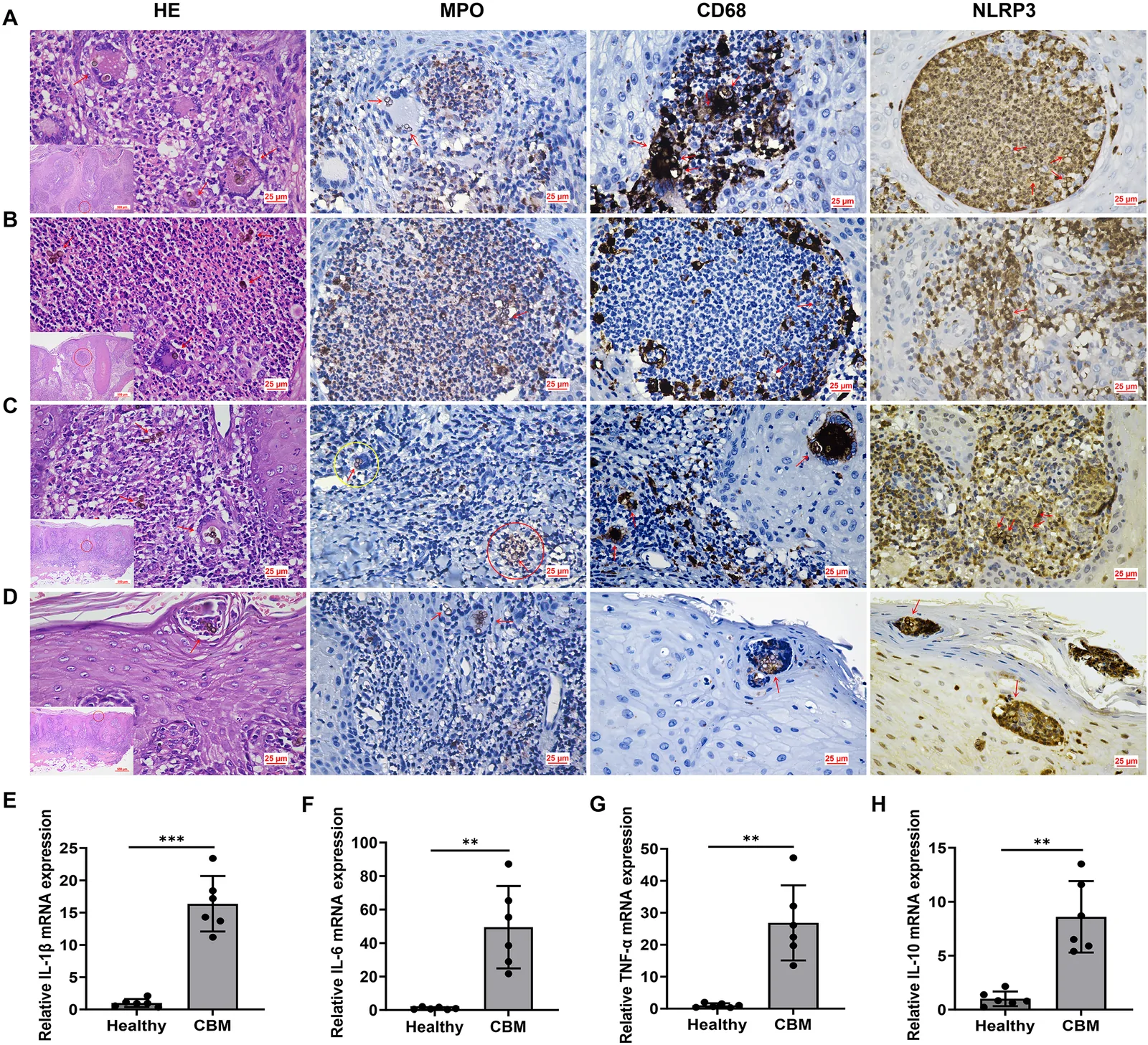
Defective human macrophage killing and multinucleated giant cell formation sequester muriform cells from neutrophils, with robust NLRP3 expression and cytokine heterogeneity **(A–D)** Histopathological and immunohistochemical analysis of skin lesions from three patients with chromoblastomycosis. Red arrows indicate muriform cells (either within multinucleated giant cells or extracellularly). **(A)** Patient 1; **(B)** Patient 2; **(C, D)** Patient 3. For each patient, the panels from left to right show H&E staining and immunohistochemistry (IHC) for MPO (brown, highlighting human neutrophils), CD68 (brown, highlighting human macrophages), and NLRP3 (brown, highlighting NLRP3+ inflammatory cells). (Magnification: 400×; scale bar: 25 µm). **(E–H)** Relative mRNA expression levels of inflammatory cytokines in healthy (n = 6) and CBM (n = 6) tissues: **(E)** IL-1β, **(F)** IL-6, **(G)** TNF-α, **(H)** IL-10. Data are presented as mean ± SD. **p < 0.01; ***p < 0.001 (two-tailed Student’s t-test).

In patients with CBM, H&E staining revealed focally distributed, extensive mixed inflammatory cell infiltrates throughout the dermis and subcutaneous region (**Figures 1A–C**; HE staining). These infiltrates aggregated to form micro-abscesses, accompanied by the presence of multi-nucleated giant cells (**Figures 1A–D**; HE staining). Within these micro-abscesses, pigmented, thick-walled muriform cells with single or transverse septa were observed, both extracellularly and within the multi-nucleated giant cells (**Figures 1A–D**). Immunostaining for MPO revealed that in the lesional skin of CBM patients, although marked recruitment of MPO+ neutrophils was observed to participate in the encapsulation of muriform cells, multinucleated giant cell formation partially sequestered some muriform cells from neutrophils (**Figures 1A–D**; MPO staining). By contrast, CD68 staining revealed that the CD68+ macrophages were predominantly recruited into the infectious foci containing muriform cells (**Figures 1A–D**; CD68 staining). Although these macrophages participated in phagocytosis, encapsulation—via multi-nucleated giant cell formation—and even trans-epidermal elimination of muriform cells (**Figures 1A–D**), they ultimately failed to clear the fungi. Indeed, the presence of budding muriform cells within multinucleated giant cells reflects pathogen viability and ongoing proliferation, a finding that is consistent with the chronic and invasive nature of CBM skin lesions. (**Figure 1C**; MPO and CD68 staining; **Supplementary Figures 1A–F**). Simultaneously, immunostaining for NLRP3, a key inflammatory receptor for macrophages, demonstrated its robust and consistent expression within the majority of inflammatory infiltrates at the same infection foci, where the muriform cells resided (**Figures 1A–D**; NLRP3). Consistent with this finding, RT-PCR analysis further demonstrated significantly elevated mRNA levels of both pro-inflammatory cytokines (IL-1β, IL-6, TNF-α) and the anti-inflammatory cytokine IL-10 in CBM patients compared to healthy controls (Student’s t test, p < 0.001, p = 0.005, p = 0.003, p = 0.002, respectively; **Figures 1E–H**).

### 3.2 *In vitro* induction efficiently converts saprophytic *F. pedrosoi* into muriform cells at a high yield

Following a 7 to10-day slide culture on PDA medium at 28 °C, the strain exhibited *Fonsecaea*-type or *Rhinocladiella*-type conidiation under light microscopy, consistent with the typical morphological features of the genus *Fonsecaea* (**Supplementary Figure 2A**). In contrast, after culture in ATCC medium 830 at 36 °C for 60 days, the majority of the saprophytic hyphal fragments of *F. pedrosoi* transformed into dark brown, swollen, thick-walled fungal cells with transverse septa, i.e., muriform cells. After dissociation and removal of the long hyphal fragments by filtration, the purity of the dispersed muriform cells reached approximately 85%, which was calculated as the mean percentage of muriform cells relative to total cells counted across at least 10 randomly chosen microscopic fields (**Supplementary Figures 2B, C**).

### 3.3 Muriform cell inoculation induces chronic CBM in WT mice with human-like pathology

**Figure 2:**
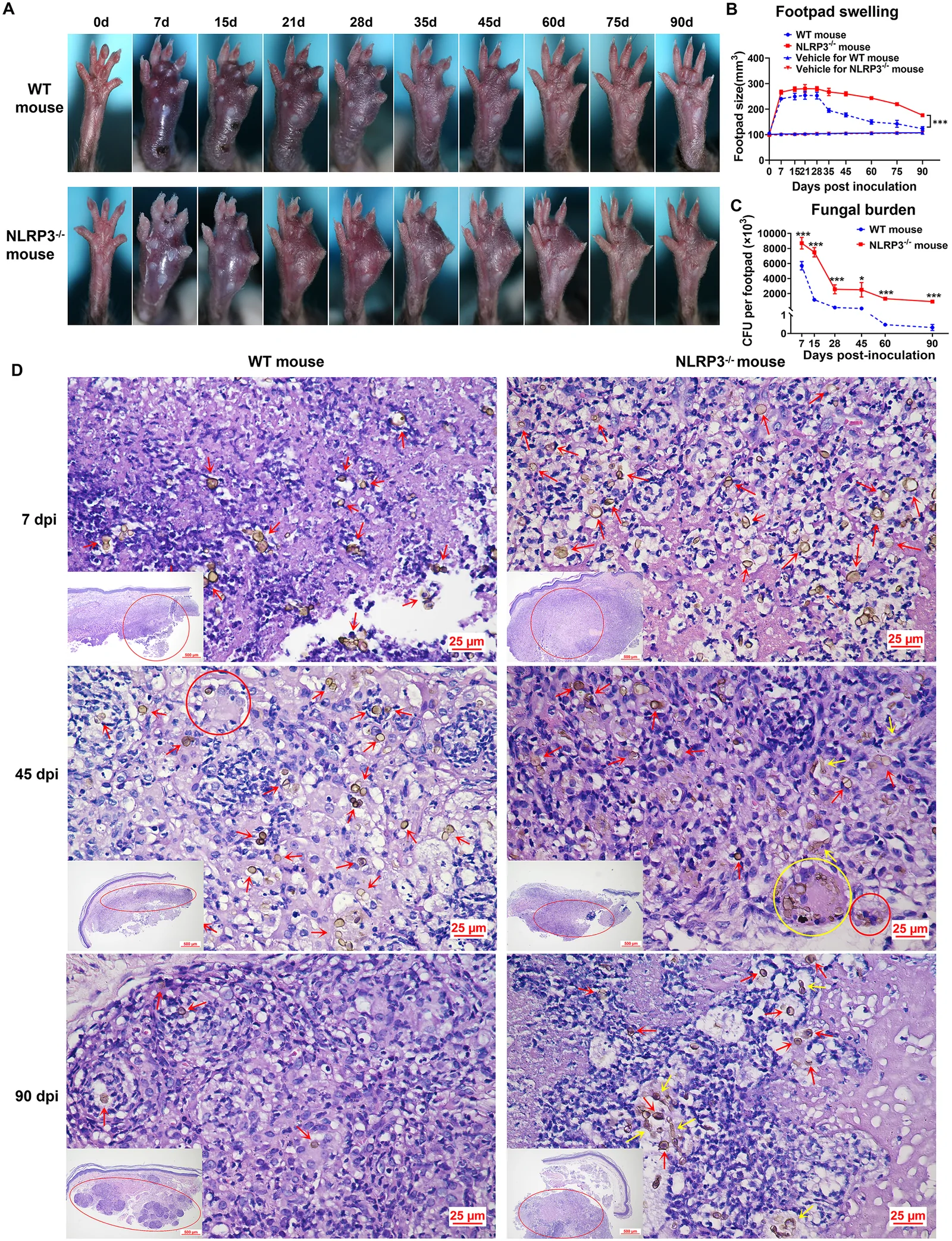
NLRP3 deficiency exacerbates disease severity and facilitates hyphal transition in a chronic chromoblastomycosis mouse model: **(A–D)** WT and NLRP3–/– mice were subcutaneously inoculated with 100 µL of muriform cell suspension (1.5×10^8^ cells/mL) in the footpad: **(A)** Representative images of infected footpads from the same WT and NLRP3–/– mice, taken at the indicated time points. **(B)** Footpad swelling was measured with a caliper over a 90-day period (n = 3 per group). **(C)** Fungal loads in whole infected footpads of WT and NLRP3–/– mice were determined at the indicated time points, and expressed as colony forming units (CFU) (n = 4 per group at each time point). **(D)** Histopathological analysis of infected footpads at 7, 45, and 90 dpi (H&E staining; magnification: ×400; scale bar: 25 µm). Red arrows indicate *F. pedrosoi* muriform cells; yellow arrows indicate hyphal forms. Red circle denotes multinucleated giant cells phagocytosing muriform cells, and yellow circle denotes transitional hyphae within the multinucleated giant cell. Data are presented as mean ± SD (n = 3 for B; n = 4 for C). Repeated measures ANOVA was used for **(B)**, and two-tailed Student’s t-test for **(C)** (WT *vs*. NLRP3–/– group at the same time point, ***p < 0.001).

In WT mice subcutaneously inoculated with *F. pedrosoi* muriform cells, footpad swelling appeared shortly after inoculation and remained significantly elevated compared to the control group throughout the 90-day observation period (Repeated-measures ANOVA, p < 0.001; **Figures 2A, B**). Briefly, this lesion progressed to ulceration and necrosis with dark purulent exudate within 7 dpi, and the footpad size peaked between 21 and 28 dpi (**Figures 2A, B**). Thereafter, clinical symptoms were significantly ameliorated, as evidenced by the gradual decrease of footpad swelling and the healing of ulcerations, and by 90 dpi, the footpad size was only marginally higher than the pre-inoculation level (**Figures 2A, B**). Meanwhile, fungal loads (CFU) in the infected footpad decreased progressively at the indicated time points over the 90-day observation period (**Figure 2C**). Despite this reduction, fungal cultures of the infected footpads remained positive at 90 dpi.

**Figure 3:**
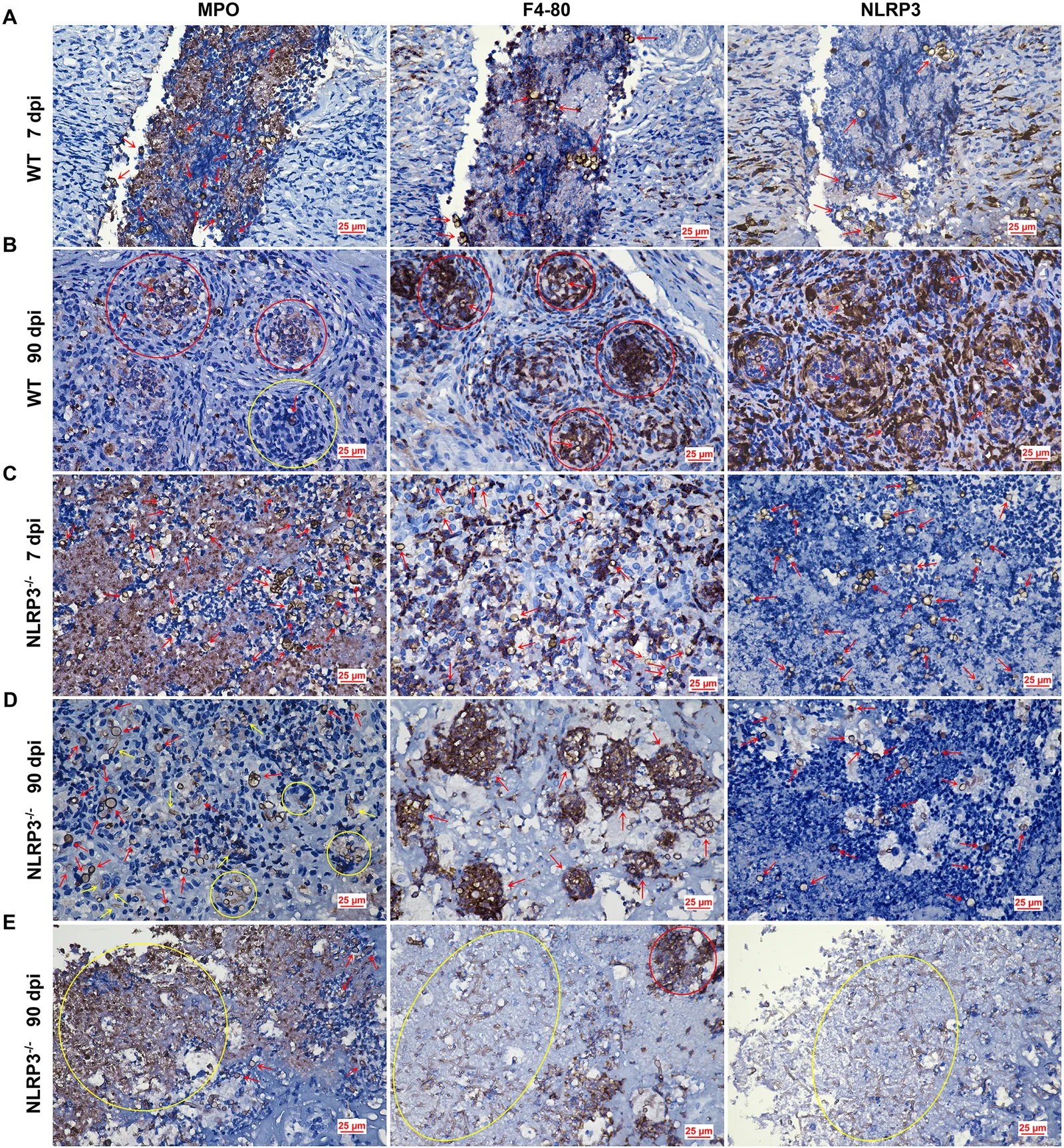
Macrophage and neutrophil infiltration in footpad lesions of WT and NLRP3–/– mouse chromoblastomycosis models: **(A–E)** IHC analysis of the footpad lesional tissues in WT and NLRP3–/– mice at 7 and 90 days post-inoculation with muriform cells. **(A)** WT, 7 dpi: muriform cell-rich foci with dense macrophage infiltration; **(B)** WT, 90 dpi: persistent muriform cell-rich foci; **(C)** NLRP3–/–, 7 dpi: muriform cell-rich foci; **(D)** NLRP3–/–, 90 dpi: muriform cell-rich foci; **(E)** NLRP3–/–, 90 dpi: transitional hyphae-rich foci devoid of macrophage accumulation. Red arrows and red circles indicate muriform cells, and yellow circles indicate transitional hyphal aggregates. Left panel shows IHC staining for MPO (brown, highlighting murine neutrophils), middle panel shows IHC staining for F4/80 (brown, highlighting murine macrophages), and right panel shows IHC staining for NLRP3 (brown and yellowish, indicating NLRP3+ inflammatory cells). (Magnification: 400×; scale bar: 25 µm).

H&E staining revealed that mixed inflammatory cells densely infiltrated the subcutaneous foci containing muriform cells (**Figure 2D**; WT). The distribution of inflammatory cells shifted gradually from a diffuse pattern at 7 dpi to a focal pattern at 90 dpi, a change that paralleled the clinical amelioration of symptoms and the decreased fungal loads in tissue (**Figure 2D**; WT). However, in line with the positive fungal cultures at 90 dpi, the inoculated muriform cells were not completely eliminated and remained encapsulated within multinucleated giant cells (**Figure 2D**; WT; 90 dpi). This finding mirrors the histological features and chronicity observed in human CBM. Furthermore, MPO and F4/80 immunostaining at 7 and 90 dpi demonstrated that although MPO+ neutrophils were markedly recruited and surrounded muriform cells in early purulent lesions (7 days post-inoculation), in late granulomatous lesions (90 days post-inoculation), MPO+ neutrophils only surrounded a subset of muriform cells (**Figures 3A, B**; MPO staining). By contrast, F4/80+ macrophages actively surrounded and encapsulated the majority of these fungal elements but failed to eliminate them, leading to multinucleated giant cell formation, mirroring human CBM pathology (**Figures 3A, B**; F4/80 staining; **Figure 2D**; WT mice, 90 dpi). Moreover, NLRP3+ inflammatory cells were observed in the vicinity of the muriform cells, displaying a distribution pattern similar to that of F4/80+ macrophages, especially at the later stage of infection (90 dpi) (**Figures 3A, B**; NLRP3 staining).

### 3.4 NLRP3 deficiency exacerbates murine chromoblastomycosis and facilitates hyphal transition, histologically manifested as prominent macrophage accumulation in muriform-rich foci and robust neutrophil infiltration into hyphal regions

Following inoculation with *F. pedrosoi* muriform cells, NLRP3–/– mice developed rapid footpad swelling that remained significantly higher than that of WT mice and sham-inoculated controls throughout the observation period (Repeated-measures ANOVA, p < 0.001; **Figures 2A, B** ). Specifically, footpad volume in this group peaked at 7 dpi, exhibited a sustained plateau until 35 dpi, and then gradually declined (**Figure 2B**). Notably, even at the experimental endpoint 90 dpi, swelling remained significantly elevated above the pre-inoculation level (**Figure 2B**). Meanwhile, the fungal burden (CFU) in the footpads of NLRP3–/– mice remained consistently higher than that in the WT group at each indicated time point assessed throughout the study (Student’s t test, all p < 0.01; **Figure 2C**), with a rapid and marked reduction observed within the first 28 days followed by a slower decline thereafter until 90 dpi (**Figure 2C**).

Histological examination at 7 dpi (early stage) showed that the inoculated muriform cells in NLRP3–/– mice were surrounded by a dense mixed inflammatory infiltrate, comparable to that in the WT group (**Figure 2D**, NLRP3–/–; 7 dpi). Strikingly, a marked divergence emerged at the later stages. In WT mice, the fungal elements remained as inoculated muriform cells within the infectious foci at all time points examined (**Figure 2D**; WT). However, in NLRP3–/– mice at 45 and 90 dpi, budding from muriform cells and hyphal transition were observed, both within multinucleate giant cells and extracellularly (**Figure 2D**; NLRP3–/–; 45 and 90 dpi). For NLRP3–/– mice, MPO staining revealed that although MPO+ neutrophils were markedly recruited and surrounded muriform cells in early purulent lesions (7 dpi) (**Figure 3C**; MPO staining), they were predominantly recruited to hyphal regions at the later stage of infection (90 dpi) (**Figure 3E** MPO staining). In contrast, F4/80 staining showed that F4/80+ macrophages were concentrated specifically in foci containing muriform cells (**Figures 3A–D**; F4/80 staining), whereas they were largely absent from regions predominated by hyphal aggregates at 90 dpi (**Figure 3E**; F4/80 staining). In addition, the absence of NLRP3 immunostaining confirmed the gene knockout in NLRP3–/– mice (**Figures 3C–E**; NLRP3 staining).

### 3.5 In WT mice, following the transition from a pyogenic to a chronic granulomatous pattern, the levels of pro-inflammatory cytokines (IL-1β, IL-6, IL-17A, and TNF-α) within the infectious foci showed a declining trend, whereas in NLRP3-deficient mice, these cytokines remained elevated in an NLRP3-independent manner

At all time points examined (7, 45, and 90 days post-inoculation), IL-1β, IL-17A, IL-6, and TNF-α were detectable in both WT and NLRP3–/– mice infected with *F. pedrosoi* muriform cells, whereas IL-10 was undetectable throughout the observation period. In WT mice, as the infectious foci transitioned from a purulent pattern (day 7 post-inoculation) to a granulomatous pattern (day 90 post-inoculation), the levels of IL-1β, IL-17A, IL-6, and TNF-α appeared to decline in parallel over time. In NLRP3–/– mice, IL-1β, IL-17A, IL-6, and TNF-α remained detectable across all time points, without an apparent declining trend. Of note, IL-1β and IL-17A levels were sustained even in the absence of NLRP3 (**Supplementary Figure 3**).

### 3.6 Chitin accumulation on muriform cells attenuates NLRP3-dependent IL-1β secretion and the broader cytokine response in mBMDMs

**Figure 4:**
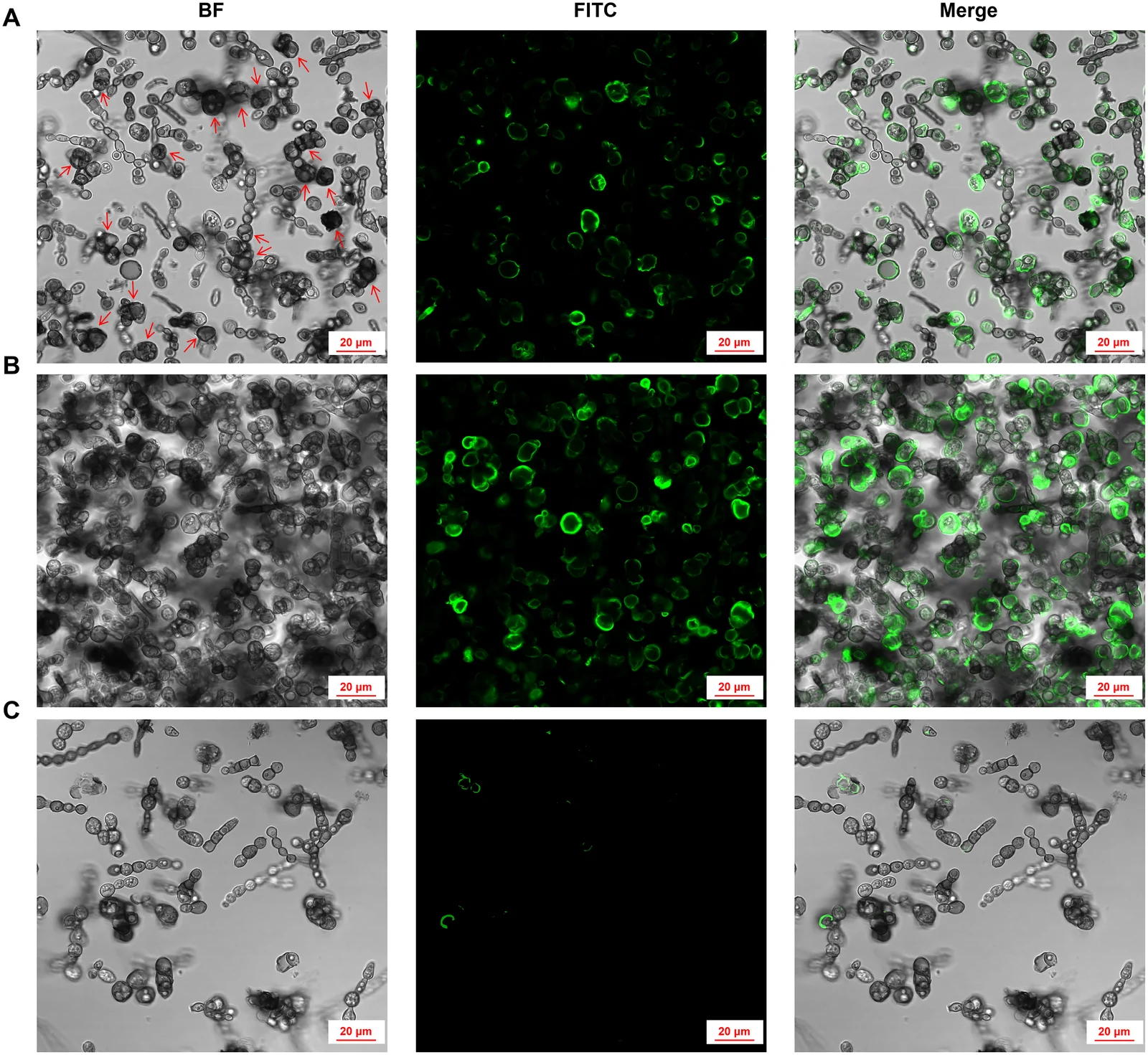
FITC-WGA staining reveals chitin distribution on the surface of muriform cells and at the tips of growing hyphae: **(A, B)** *In vitro*-induced muriform cells after culture in ATCC 830 medium for 35 days **(A)** and 60 days **(B)**. **(C)** After culture in ATCC 830 medium for 35 days, 1.0 × 10^8^ muriform cells (including non-transformed hyphal fragments) were treated with chitinase (25 U in 5 mL PBS) at 37 °C overnight. Left panel: bright-field images showing cell morphology. Red arrows indicate the swollen, thick-walled fungal cells with transverse septa-the muriform cells; middle panel: FITC-WGA fluorescence (excitation/emission: 488 nm/510–540 nm); right panel: merged images. (Magnification: 630×, oil immersion; Scale bar: 20 μm).

After 35 or 60 days of culture in ATCC 830 medium, confocal microscopy revealed that FITC-WGA specifically localized to the surface of most muriform cells with visible septa (**Figures 4A, B**; middle and right panels). In contrast, minimal binding was observed on hyphal structures, except at the tips of growing hyphae (**Figures 4A, B**, right panel). Following chitinase digestion, WGA binding on the surface of muriform cells was markedly reduced to near-undetectable levels (**Figure 4C**).

**Figure 5:**
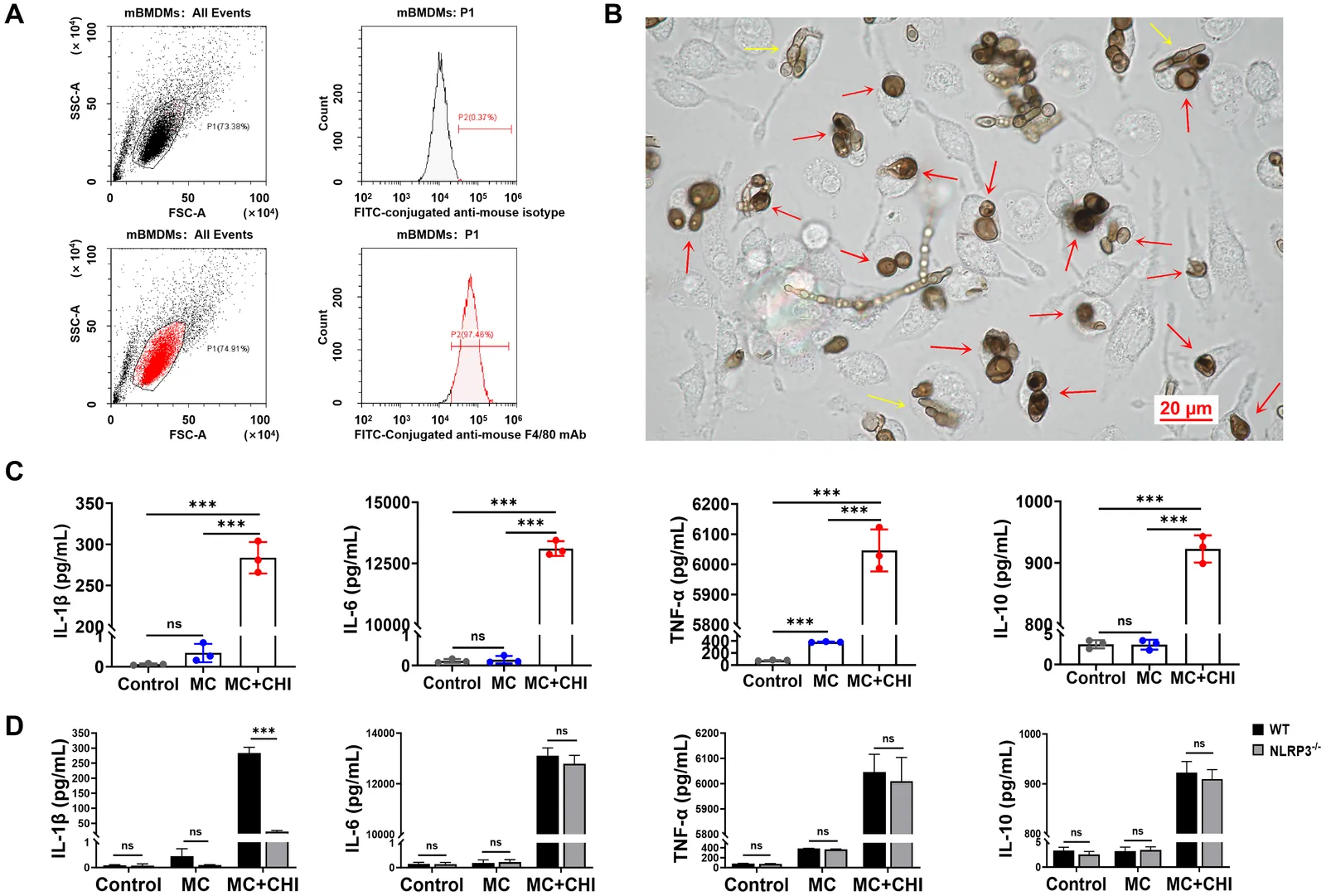
Surface chitin on muriform cells suppresses NLRP3-dependent IL-1β and the broader cytokine response in mBMDMs: **(A)** Representative flow cytometry plots showing the gating strategy for mature mouse bone-marrow-derived macrophages (mBMDMs). Cells were first gated on P1 (singlets, FSC-A vs. SSC-A), followed by gating on P2 (F4/80⁺ cells) from the P1 gate. Numbers indicate the percentage of cells in the parent gate. **(B)** Representative bright-field microscopy images showing phagocytosis of heat-inactivated muriform cells by WT and NLRP3–/– mBMDMs. Red and yellow arrows indicate muriform cells (with single or transverse septa) and non-transformed hyphal fragments, respectively, both of which have been phagocytosed by macrophages. (Magnification: 630×, oil immersion; scale bar: 20 μm) **(C)** Quantification of cytokine levels in WT mBMDMs under indicated conditions. **(D)** Comparison between WT and NLRP3⁻/⁻ mBMDMs under indicated conditions. Data are presented as mean ± SD (n = 3). Statistical significance was determined by one-way ANOVA followed by *post hoc* LSD-t test for **(C)** and Two-tailed Student’s-t test for **(D)**. (Pairwise comparisons among groups, ***p < 0.001. ns, not significant).

Prior to stimulation, the purity of F4/80+ mBMDMs reached 97.46% (> 95%), as determined by flow cytometry (**Figure 5A**). Following co-culture for 14 h, mBMDMs derived from both WT and NLRP3–/– mice readily phagocytosed heat-inactivated muriform cells, irrespective of chitinase pretreatment (**Figure 5B**). Simultaneously, CBA analysis of the supernatants demonstrated that intact muriform cells induced the secretion of TNF-α alone to a comparable extent in both WT and NLRP3–/– mBMDMs, with no detectable levels of IL-1β, IL-6 or IL-10 (**Figures 5C, D**). In contrast, compared with untreated cells or those stimulated with intact muriform cells, treatment with chitinase-digested muriform cells significantly increased the release of IL-6, IL-10, and TNF-α in WT and NLRP3–/– mBMDMs (ANOVA with *post hoc* LSD-t test, all p < 0.01; **Figures 5C, D**), with no significant difference observed between the two genotypes. Notably, upon stimulation with chitinase-digested muriform cells, IL-1β secretion was significantly reduced in NLRP3–/– mBMDMs compared to WT counterparts, (Student’s t test, p < 0.001; **Figure 5D**), whereas a mild increase relative to untreated cells or those stimulated with intact muriform cells was still observed in the NLRP3–/– genotype (Student’s t test, p = 0.01; **Figure 5D**).

Furthermore, IL-18 was undetectable in the supernatants of both WT and NLRP3-deficient BMDMs, irrespective of stimulation. In parallel, IL-18 mRNA levels remained uniformly low across all conditions in WT cells. In contrast, chitinase-pretreated muriform cells induced a marked upregulation of IL-1β mRNA compared with untreated cells or those exposed to intact muriform cells (ANOVA with *post hoc* LSD-t test, p<0.001; **Supplementary Figure 5**).

These findings indicate that chitin accumulation on the surface of muriform cells broadly suppressed the production of both pro- and anti-inflammatory cytokines in macrophages, with IL-1β being specifically attenuated in an NLRP3-dependent manner.

### 3.7 Mechanistically, this chitin accumulation partly suppresses the NLRP3 priming and downstream signaling activation

**Figure 6:**
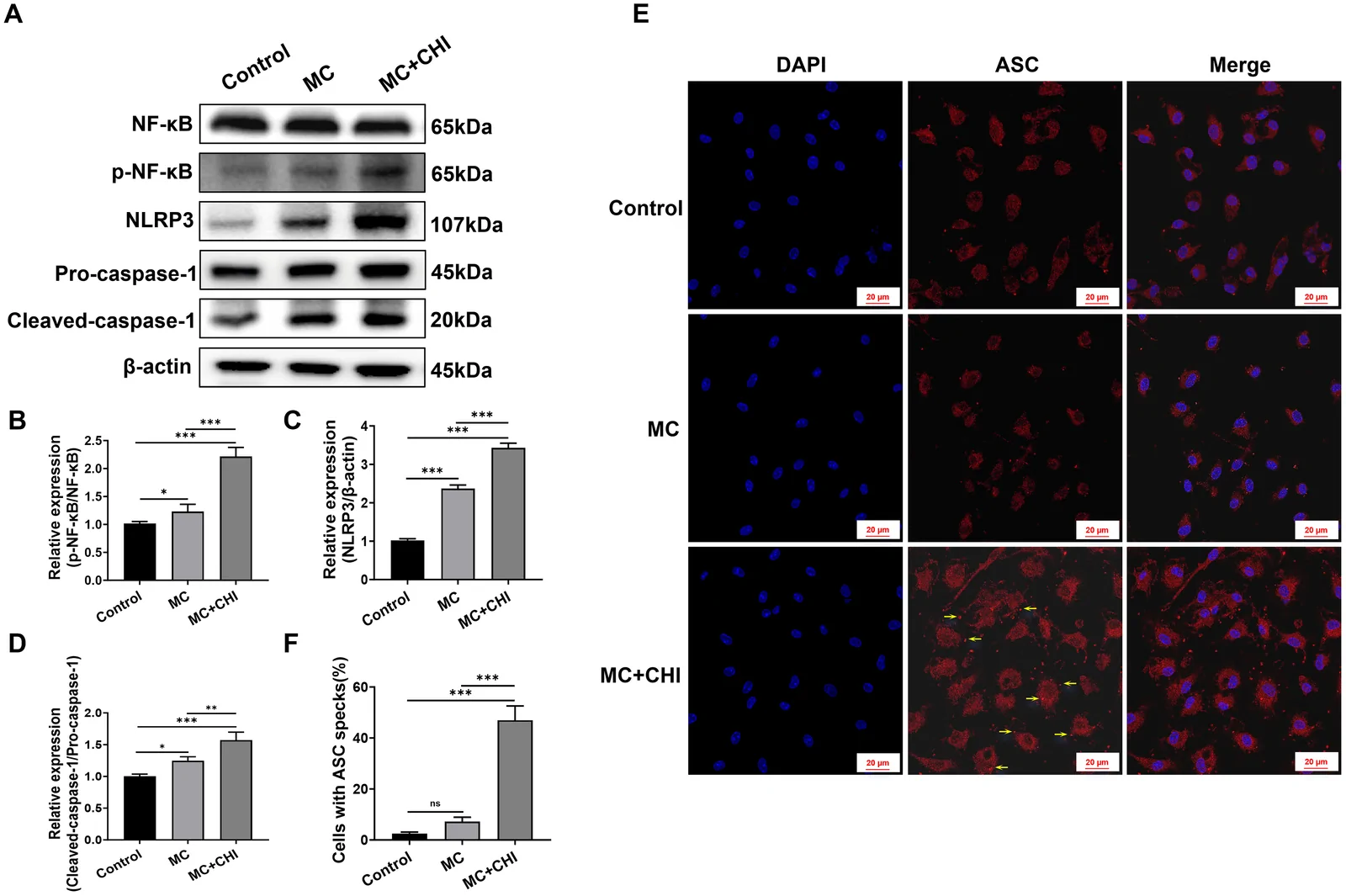
Chitin accumulation partly suppresses the NLRP3 priming and downstream signaling activation: **(A)** Western blot analysis of NF-κB phosphorylation (p-NF-κB), NLRP3 expression, and Caspase-1 cleavage in mouse BMDMs stimulated with intact (MC) or chitinase-pretreated muriform cells (MC+CHI) for 14 h. Control: untreated mBMDMs. Signals were visualized using ECL chemiluminescence substrate. **(B, C, F)** Densitometric quantifications of p-NF-κB/NF-κB **(B)**, NLRP3/β-actin **(C)**, and cleaved Caspase-1/pro-Caspase-1 **(F)**. **(D)** Immunofluorescence staining of ASC specks (red) in BMDMs. Nuclei were stained with DAPI (blue). Yellow arrows indicate ASC specks. (Magnification: 400×; scale bar: 20 μm) **(E)** Percentage of cells with ASC specks. **(B, C, E, F)** Data are presented as mean ± SD (n = 3). Statistical significance was determined by one-way ANOVA followed by *post hoc* LSD-t test. (Pairwise comparisons among groups, *p < 0.05; **p < 0.01; ***p < 0.001. ns, not significant).

Western blotting with densitometric analysis was performed to assess the activation of the NLRP3 inflammasome pathway. Upon stimulation with intact muriform cells, the ratio of phosphorylated NF-κB (p-NF-κB) to total NF-κB (a key trigger for NLRP3 priming), and NLRP3 expression in mBMDMs were significantly up-regulated compared to the control group (ANOVA with *post hoc* LSD-t test, p = 0.021 for p-NF-κB/NF-κB, p < 0.001 for NLRP3; **Figures 6A–C**). Notably, these effects were further enhanced significantly following stimulation with chitinase-digested muriform cells compared to muriform cell stimulation group (ANOVA with *post hoc* LSD-t test, p < 0.001 for both p-NF-κB/NF-κB and NLRP3; **Figures 6A–C**). Additionally, confocal microscopy demonstrated that a substantial increase in ASC speck formation, a critical indicator of NLRP3 inflammasome activation characterized by a single, dense, cytoplasmic punctate structure, was observed in mBMDMs stimulated with chitinase-digested muriform cells compared to those observed in the control group or the muriform cell-stimulated group (ANOVA with *post hoc* LSD-t test, P < 0.001; **Figures 6D, E**). By contrast, ASC speck formation was not significantly altered in mBMDMs stimulated with intact muriform cells compared with the control group (**Figures 6D, E**). Consistently, while the ratio of cleaved caspase-1/pro-caspase-1 was mildly up-regulated in mBMDMs stimulated with intact muriform cells compared to the control group (ANOVA with *post hoc* LSD-t test, p = 0.012), a greater increase was observed in chitinase-treated muriform cell group (ANOVA with *post hoc* LSD-t test, p < 0.001 vs. control group, p = 0.003 vs. intact muriform cell group; **Figures 6A, F**). Collectively, these findings suggest that chitin accumulation on muriform cells contributed to suppressing the NLRP3 priming and downstream activation process in mBMDMs.

## 4 Discussion

Our previous work, along with other reports, agrees that neutrophils are key effector cells against the parasitic muriform form of *F. pedrosoi*, as they produce ROS and undergo NETosis to eliminate these fungal elements (21–23). In the present study, we found that while MPO+ neutrophils were significantly recruited to CBM lesions and surrounded partial muriform cells, CD68+ macrophages were also coordinately recruited to these lesions. There, macrophages contributed to the host immune response through phagocytosis, encapsulation, and transepidermal elimination; however, they failed to eradicate the fungal elements. Instead, these macrophages fused into multinucleated giant cells, within which budding and proliferation of the parasitic form still occurred. These findings suggest that macrophages may exert a pathogenic rather than protective function, and partly sequester muriform cells from neutrophils, potentially driving the chronicity of CBM lesions.

Notably, significant and consistent expression of NLRP3 was detected in inflammatory cells recruited to these infectious foci containing muriform cells. This raises the possibility that the NLRP3 inflammasome, a key inflammatory signaling pathway in macrophages (15, 16, 19, 20), is primed, thereby facilitating its subsequent activation during the infection. RT-PCR analysis performed on the same CBM tissues consistently revealed a marked up-regulation in the transcriptional expression of the pro-inflammatory cytokines IL-1β, TNF-α, and IL-6, while the anti-inflammatory cytokine IL-10 was also significantly elevated. We therefore speculate that NLRP3 inflammasome activation in infectious foci, especially within macrophages, would further drive IL-1β cleavage and maturation. The released IL-1β then works together with other mentioned cytokines to recruit and activate immune effector cells, thus enhancing host antifungal immunity (17). Nevertheless, the presence of NLRP3 expression in infectious foci and its contribution to the host inflammatory response are not sufficient to alter the chronic course of CBM infection, as evidenced here by the ability of muriform cells to circumvent host immune clearance and proliferate by budding inside multinucleated giant cells, thereby allowing their long-term persistence in tissue. This ultimately leads to the further progression of CBM, a process driven in part by the production of immunosuppressive cytokines, including IL-10 (24).

To further explore the impact of NLRP3 on the inflammatory cell infiltration profile and disease outcome, we have established the CBM models in WT and NLRP3–/– mice via subcutaneous footpad injection with *F. pedrosoi* muriform form cells. Here we have further demonstrated that in WT mice, as the infectious foci transitioned from a purulent pattern (day 7) to a granulomatous pattern (day 90), the levels of IL-1β, IL-17A, IL-6, and TNF-α declined in parallel. Histologically, MPO+ neutrophils were abundantly recruited around muriform cells in early purulent lesions (day 7). In contrast, at day 90, MPO+ neutrophils surrounded only a subset of muriform cells, whereas F4/80+ macrophages enveloped the majority of fungal elements without eliminating them, resulting in multinucleated giant cell formation—a feature recapitulating human chromoblastomycosis.

By contrast, in NLRP3–/– mice, IL-1β and IL-17A were secreted in an NLRP3-independent manner and exhibited no apparent declining trend across the time points examined. Furthermore, as muriform cells increasingly budded and gradually transitioned to hyphae both inside and outside multinucleated giant cells in NLRP3–/– lesions, MPO+ neutrophils were predominantly recruited to hyphal regions, while F4/80+ macrophages remained relatively confined to areas containing muriform cells. Given that the self-resolving subcutaneous infection induced by *F. pedrosoi* hyphae in NLRP3–/– mice has been documented previously (25), these findings suggest that neutrophil infiltration driven by an IL-1β/IL-17 axis—operating independently of the NLRP3 inflammasome—may contribute to the elimination of hyphal elements, potentially via ROS production or NETosis. On the other hand, the concentrated recruitment of NLRP3–/– macrophages to areas harboring muriform cells was insufficient to eliminate these fungal elements, and was instead accompanied by multinucleated giant cell formation, as well as budding of muriform cells and their subsequent transition to hyphae. This coexistence of hyphae and muriform cells within the lesions may contribute to the chronic progression of infection and persistent neutrophil-mediated inflammation.

Based on these findings, although NLRP3 expression in macrophages did not prevent the chronic outcome of footpad infection by the muriform form in WT mice, it actively participated in inhibiting the budding and hyphal transition of these fungal elements. Thus, NLRP3 plays a protective role against subcutaneous invasive infection caused by CBM pathogens, and also modulates, at least in part, the infiltration profile of inflammatory cells within the lesions. Notably, activation of the NLRP3 signaling pathway contributes to the maintenance of muriform morphology and the formation of granulomatous pathological patterns. However, for chaetothyriales fungi causing CBM, the present study corroborates that maintaining the muriform phenotype may represent an adaptive strategy to interfere with NLRP3-mediated inflammatory responses, thereby evading macrophage killing and facilitating long-term tissue colonization. Given that macrophages are the key immune cells interacting with muriform cells in late-stage lesions, the inability of these phagocytes to eliminate internalized fungal elements is a pivotal factor driving chronic progression. We therefore propose that further investigation into the potential mechanisms by which muriform cells suppress NLRP3-related signaling pathways in host macrophages will be crucial for elucidating the persistence of CBM.

Furthermore, the present study independently confirmed that chitin components were predominantly localized on the surface of induced *F. pedrosoi* muriform cells, but not on the hyphal surface, which was consistent with our prior findings (21, 22). Under conditions of chitin accumulation, we demonstrated that the muriform cells can only induce limited production of TNF-α. Following enzymatic degradation with chitinase, muriform cells potentially stimulated the secretion of pro-inflammatory (IL-1β, IL-6, TNF-α) and anti-inflammatory (IL-10) cytokines from mBMDMs, whereas IL-18 secretion remained undetectable. Notably, IL-18 mRNA levels were uniformly very low across all stimulation conditions, whereas IL-1β mRNA was strongly induced by chitinase-pretreated muriform cells. Considering that NLRP3 inflammasome activation primarily promotes the proteolytic processing of pro-IL-1β and pro-IL-18, the very low expression of pro-IL-18 likely explains why mature IL-18 remains undetectable despite caspase-1 activation. Additionally, although our ELISA is widely used, limited assay sensitivity cannot be completely ruled out as a contributing factor. Taken together, our findings suggested that chitin accumulation on muriform cells substantially suppressed the secretion of pro-inflammatory and anti-inflammatory cytokines in host macrophages, reflecting the immunologically inert nature of this molecule.

Furthermore, we confirmed that IL-1β production and secretion by mBMDMs stimulated with chitinase-treated muriform cells occurred in an NLRP3-dependent manner, whereas the secretion of IL-6, TNF-α, and IL-10 showed no significant correlation with NLRP3 knockout. Accordingly, we infer that the chitin-mediated inhibition of IL-1β secretion is primarily achieved through interference with the activation of the NLRP3 signaling pathway.

Activation of the NLRP3 inflammasome typically requires two sequential steps: priming and activation (26, 27). During the priming phase, the NF-κB inflammatory signaling pathway plays a critical regulatory role (26, 27). In the present study, we demonstrate that *F. pedrosoi* muriform cells act as a weak inflammasome agonist, eliciting only mild NLRP3 activation. In contrast, chitinase-treated muriform cells, stripped of surface chitin, induce significantly enhanced NF-κB phosphorylation and NLRP3 expression in mBMDMs. These findings suggest that chitin serves as an immunosuppressive shield by attenuating the NF-κB-dependent NLRP3 priming signal. In subsequent validation during the activation phase, we further demonstrated that the “chitin accumulation” on the surface of muriform cells significantly inhibited the activation of downstream NLRP3 signaling effectors, including the formation of apoptosis-associated speck-like protein containing a CARD (ASC specks) and the proteolytic conversion of pro-Caspase-1 (p45) to its active form Caspase-1 (p20). Thus, we conclude that the “chitin accumulation” effect acts as a potent inhibitor of the NLRP3 inflammasome at both the priming and activation levels, consequently impairing the maturation and release of IL-1β.

Several limitations of this study should be acknowledged. First, the number of human samples (three CBM lesions for IHC and six for RT-qPCR) is limited due to disease rarity. Therefore, the statistical analysis regarding cytokine profiles and NLRP3 expression in human lesions are presented as preliminary observations that need further validation in larger cohorts. Second, it should be noted that while the experimental technique for inducing *F. pedrosoi* from saprophytic form into muriform cell form has been largely established, some inter-batch variability in transformation efficiency persists, with efficiencies around 85% representing our typical upper range. Obtaining muriform cells completely free of hyphal contamination remains technically challenging. Nevertheless, even a small number of hyphal fragments could influence baseline inflammatory readouts, given their known potency in activating innate immune receptors such as Dectin-1. Even at 15% proportion or less, these fragments may elevate background cytokine production, thereby potentially masking the full suppressive effect of muriform surface chitin. Third, for cytokines detection in the footpad lesion of WT and NLRP3–/– mice infected with *F. pedrosoi* muriform cells, statistical comparisons were not applied to the cytokine time-course data from footpad tissues due to the limited sample availability (n=1 per time point at 7, 45, and 90 days post-inoculation) (**Supplementary Figure 3**). Furthermore, in contrast to the relatively muted cytokine responses observed *in vitro*, elevated transcriptional levels of pro-inflammatory cytokines were consistently detected in clinical CBM tissues. This discrepancy likely arises from the temporal and cellular complexity of the *in vivo* microenvironment. While acute exposure of macrophages to chitin-rich muriform cells suppresses cytokine release, chronic lesions are characterized by sustained neutrophil infiltration and macrophage-derived chitinase activity, which collectively erode the chitin layer over time and expose immunostimulatory PAMPs. At later stages, this dynamic is further modulated by T cell exhaustion, which may dampen effector functions and promote fungal persistence (28). Collectively, these factors explain why short-term macrophage cultures cannot fully recapitulate the inflammatory milieu of chronic CBM lesions. Additionally, it should be mentioned that in our long-term experiments using a murine footpad infection model with *F. pedrosoi*, we observed that during the early phase of infection, particularly within the first 30 days post-inoculation, the mice actively resisted and cleared muriform cell inoculants. Furthermore, our preliminary experiments confirmed that if the inoculum of muriform cells per footpad was lower than 1.0×10^7^ CFU (1.0×10^8^ CFU/mL, 100 µL), the probability of the subcutaneous infection progressing toward self-healing increased significantly, accompanied by complete clearance of the pathogen. In the present study, we found that although the inoculum per footpad reached 1.5×10^7^, the fungal load in the chronically infected foci exhibiting a granulomatous pattern at 90 days post-inoculation was below 1000 CFU. This indicates that the high dose merely serves to overcome early host resistance without sustaining an unnaturally high infection load.

In summary, our work shows that while the NLRP3 inflammasome contributes to host defense against *F. pedrosoi* muriform cells, it alone is insufficient to prevent the establishment of chronic infection. This is partly attributable to the chitin component accumulated on the surface of *F. pedrosoi* muriform cells, which actively suppresses NLRP3 inflammasome activation and subsequent IL-1β release from host macrophages. Furthermore, this chitin accumulation effect significantly suppresses NF-κB phosphorylation and the secretion of pro-inflammatory cytokines, including IL-6 and TNF-α. It should be mentioned that in WT mice, as the muriform cell-residing infectious foci transitioned from a purulent pattern to a granulomatous pattern, the levels of IL-1β and IL-17A declined in parallel. These events interfere, to some extent, with macrophage activation and the subsequent recruitment of IL-1β/IL-17 axis-dependent neutrophils within the infectious foci, thereby compromising host immune clearance of the parasitic form of CBM agents and promoting the chronic progression of the infection. Accordingly, we propose that the findings in the present study, representing the tip of the iceberg, reveal an intrinsic link between the morphological transition of CBM pathogens into muriform cells and the refractory nature of the disease from the perspective of immune evasion. In our ongoing studies, we plan to establish subcutaneous infection models and perform single-cell RNA sequencing alongside flow cytometric analysis to systematically profile the dynamic changes of infiltrating immune populations.

## Data Availability

ITS sequencing data for the strain Fonsecaea pedrosoi (WH10-002) in this article can be retrieved from NCBI according to GenBank accession no. GQ420654.1. All the other original contributions are not subject to the journal’s mandatory deposition requirements, and presented in the article/**Supplementary Material**. Notably, the raw flow cytometry data are encouraged but not mandated for deposition. As they contain experimental data beyond the scope of this study, only the data directly relevant to the present study are exported and presented in the main figures and **Supplementary Material**.
