# Correction: Impaired NLRP3 inflammasome activaton by chitin underlies refractory chromoblastomycosis caused by *Fonsecaea pedrosoi* muriform cells

**DOI:** 10.3389/fimmu.2026.1955349

**Published:** 2026-08-12

**Authors:** Yao Chen, Zilu Qu, Zhaolan Xie, Xiaowen Wang, Zhongsheng Tong, Luoyao Yang, Xu Zhang, Liuqing Chen, Bilin Dong

**Affiliations:** 1 Hubei Province & Key Laboratory of Skin Infection and Immunity, Department of Dermatology, Wuhan No.1 Hospital, Wuhan, Hubei, China; 2 Hubei Provincial Clinical Research Center for Infectious Skin Diseases, Department of Dermatology, Wuhan No.1 Hospital, Wuhan, Hubei, China; 3 Central Laboratory, Wuhan Wuchang Hospital, Wuhan, Hubei, China; 4 Research Center for Medical Mycology, Department of Dermatology and Venerology, Peking University First Hospital, Beijing, China

**Keywords:** bone-marrow-derived macrophages, chitin, chromoblastomycosis, *Fonsecaea pedrosoi*, muriform cells, NLRP3 inflammasome

There was a mistake in **Table 1** as published. The forward primer of hTNF-α was inadvertently duplicated and incorrectly entered as the reverse primer sequence. Its correct forward sequence is 5’“GACAAGCCTGTAGCCCATGT” 3’. The correct primer pair was used in all the involved experiments, and we apologize for this typographical error. The corrected **Table 1** appears below.

**Table 1:** Primer sequences for human (h) or mouse (m) RT-PCR.

Gene	Forward primer (5’-3’)	Reverse primer (5’-3’)	Tm (°C)
hIL-1β	CCAAACCTCTTCGAGGCACA	GCTGCTTCAGACACTTGAGC	60
hIL-6	TTCGGTCCAGTTGCCTTCTC	CAGCTCTGGCTTGTTCCTCA	60
hTNF-α	GACAAGCCTGTAGCCCATGT	GGAGGTTGACCTTGGTCTGG	60
hIL-10	TGCAAAAGAAGGCATGCACAG	GTTCACATGCGCCTTGATGT	60
hGAPDH	TCGGAGTCAACGGATTTGGT	AGTGATGGCATGGACTGTGG	60
mIL-1β	AGAGCCCATCCTCTGTGACT	CTCTGCTTGTGAGGTGCTGA	60
mIL-18	AGAAAGCCGCCTCAAACCTT	TCAGTCTGGTCTGGGGTTCA	60
mGAPDH	TCAGGAGAGTGTTTCCTCGT	CCTTGACTGTGCCGTTGAAT	60

The original version of this article has been updated.

