# Supplementary material for: Impaired NLRP3 inflammasome activation by chitin underlies refractory chromoblastomycosis caused by Fonsecaea pedrosoi muriform cells
Source: Front Immunol. 2026 Jul 14;17:1843553. doi: 10.3389/fimmu.2026.1843553 (PMC13407277; doi:10.3389/fimmu.2026.1843553)
Supplement: Supplementary file 1 [file DataSheet1.pdf]

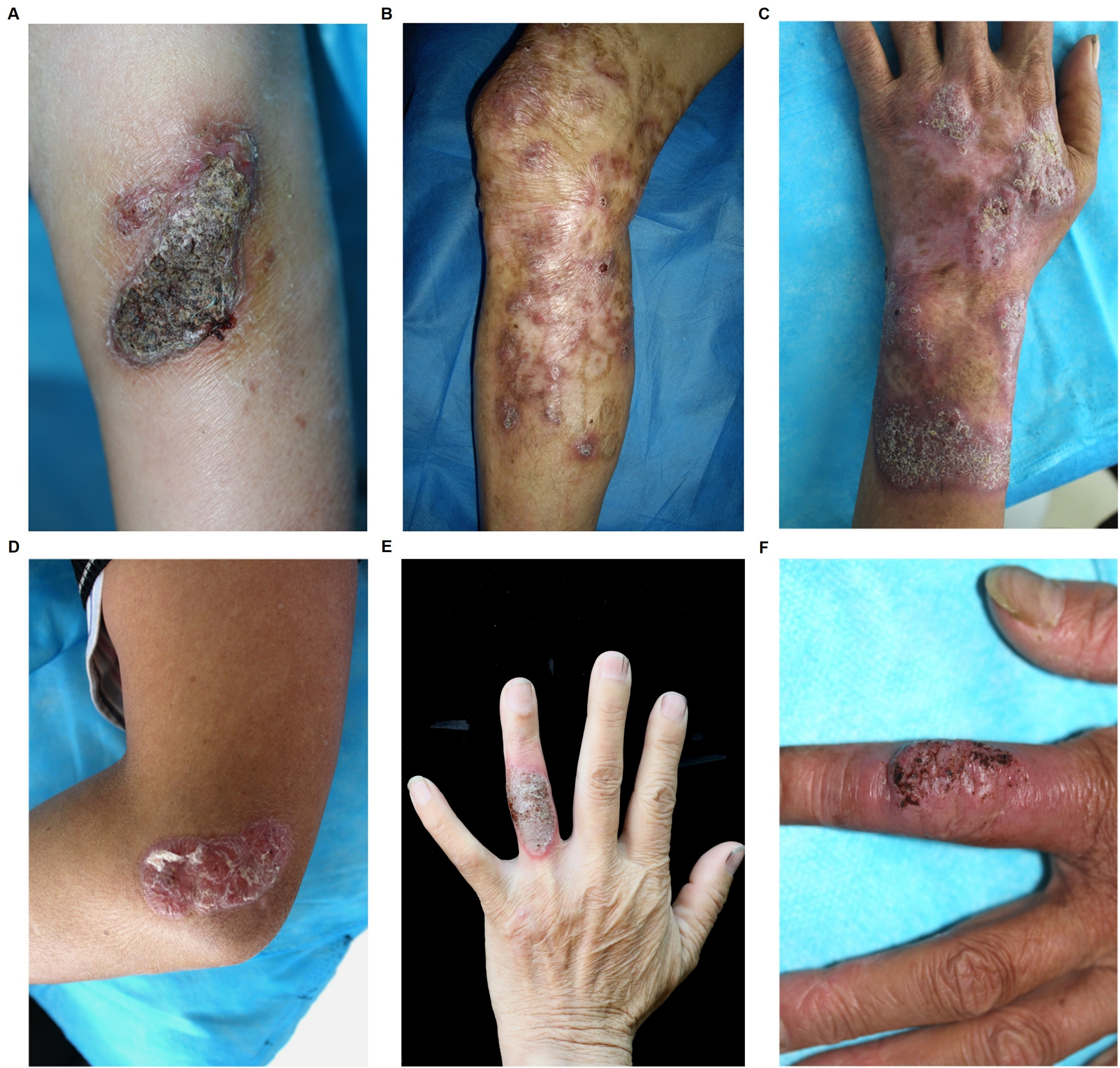
A
B
C
D
E
F

Supplementary Figure 1. Clinical presentations of patients with chromoblastomycosis (CBM): (A–F) Representative skin lesions from six patients. Diagnosis was confirmed by histological identification of muriform cells, and causative agents were identified by morphological characteristics and internal transcribed spacer (ITS) sequencing. (A) *Fonsecaea monophora* infection. Erythematous plaque with black papules and purulent discharge on the left forearm. (B) *Fonsecaea nubica* infection. Multiple dark red patches with ulceration, crusting, and atrophic scarring on the right lower extremity; some lesions show annular configuration. (C) *Fonsecaea monophora* infection. Verrucous hyperplasia with atrophic scarring and scattered black punctate crusts extending from the left palm to the forearm. (D) *Fonsecaea monophora* infection. Red verrucous hyperplastic plaque on the left elbow. (E) *Fonsecaea monophora* infection. Infiltrated red hyperplastic plaque with ulceration and black dot-like crusts on the left ring finger. (F) *Cladophialophora carrionii* infection. Red verrucous plaque with ulceration and dark crusts on the left index finger. (A–C) Corresponding histopathology shown in Figure 1.

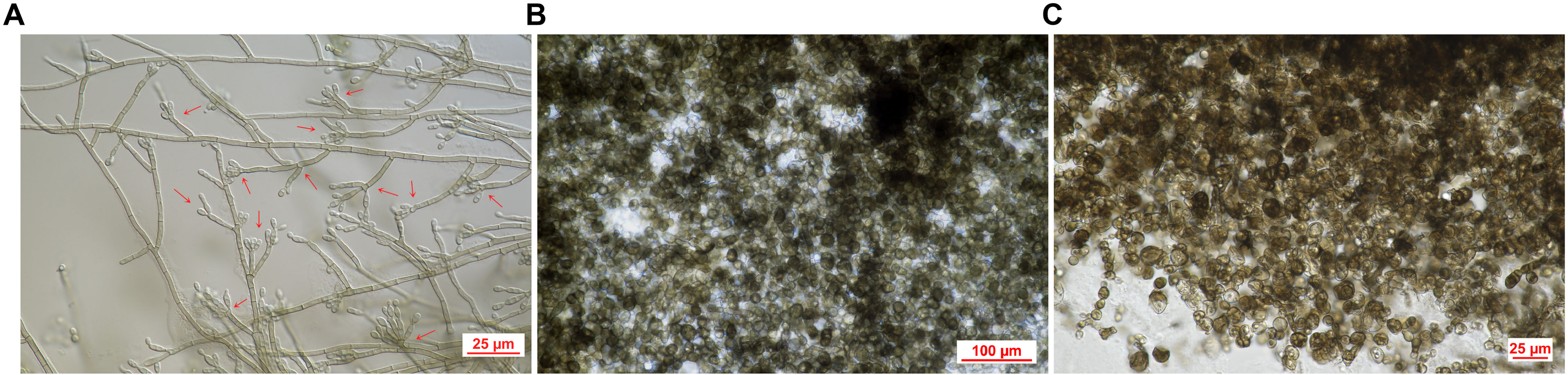
A
B
C
25 µm
100 µm
25 µm

Supplementary Figure 2. *In vitro*-induced transformation of *F. pedrosoi* (WH10-002) from saprophytic form to parasitic muriform cells: (A) The stock culture (WH10-002) was subjected to slide culture on Potato dextrose agar (PDA) at 28°C for 7-10 days and morphologically confirmed as a pure culture of the genus *Fonsecaea*. Red arrows indicate *Fonsecaea*-type or *Rhinocladiella*-type conidiation with olivaceous to dark olivaceous hyphae under light microscopy, consistent with the typical morphological features of the genus *Fonsecaea*. (oil ×600; scale bar = 25 μm). (B and C) After culture in ATCC medium 830 at 36 °C for 60 days, the majority of the saprophytic hyphal fragments of *F. pedrosoi* transformed into dark brown, swollen, thick-walled fungal cells with transverse septa, i.e., muriform cells. (B) (× 200; scale bar = 100 μm). (C) (× 400; scale bar = 25 μm)

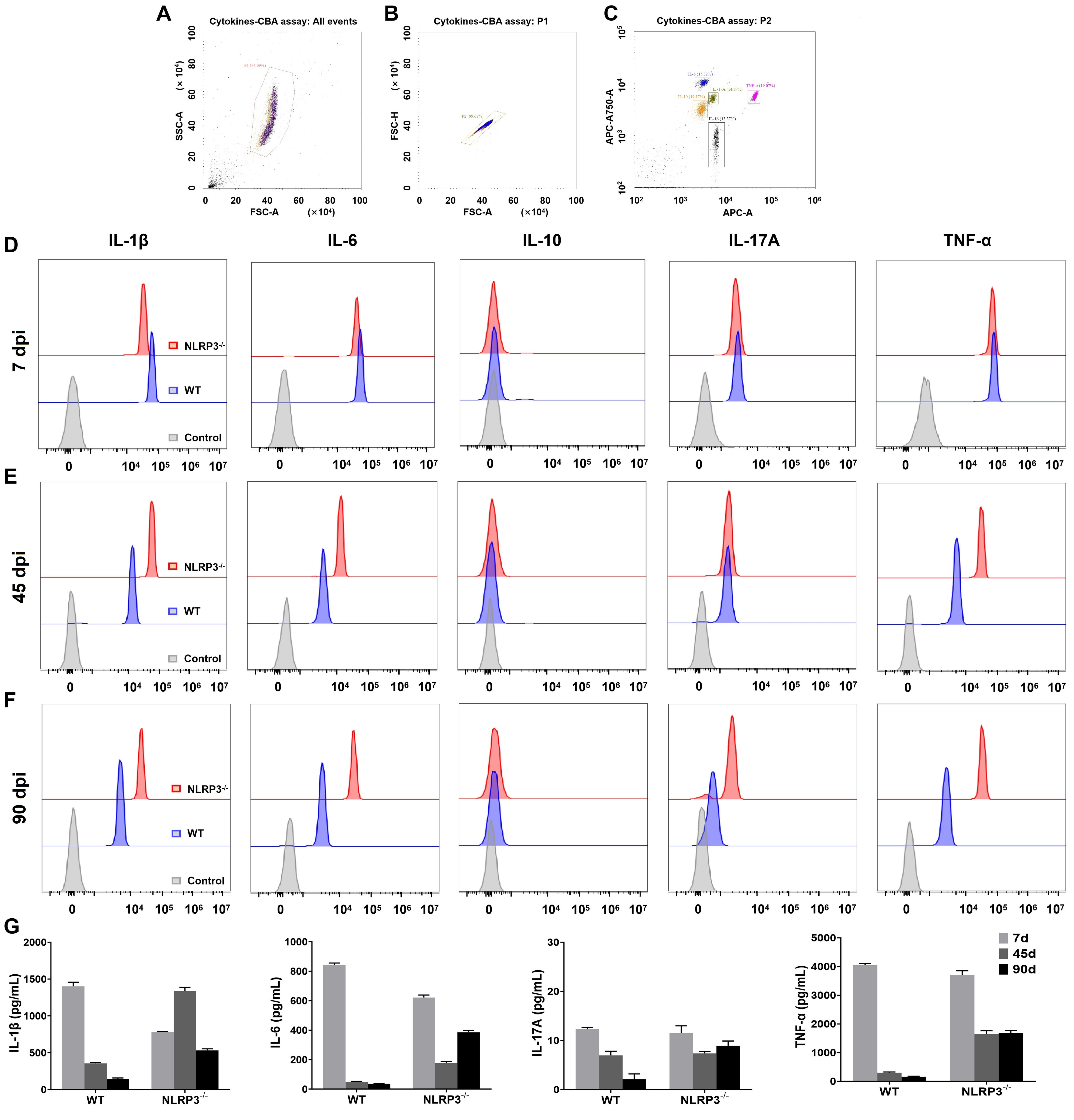

A
Cytokines-CBA assay: All events
SSC-A
FSC-A
B
Cytokines-CBA assay: P1
FSC-H
FSC-A
C
Cytokines-CBA assay: P2
APC-A750-A
APC-A
D
IL-1β
IL-6
IL-10
IL-17A
TNF-α
7 dpi
NLRP3-/-
WT
Control
E
45 dpi
F
90 dpi
G
IL-1β (pg/mL)
IL-6 (pg/mL)
IL-17A (pg/mL)
TNF-α (pg/mL)
WT
NLRP3-/-
7d
45d
90d

Supplementary Figure 3. Quantification of cytokines in mouse footpads infected with muriform cells via CBA flex set. (A and B) Single cells were gated as P1 (FSC-A *vs.* SSC-A) and then as P2 (FSC-A *vs.* FSC-H). Numbers indicate the percentage of parent-gated cells. (C) Capture antibodies immobilized on beads with distinct APC/APC-A750 fluorescence intensities enable multiplex detection of IL-1β, IL-6, IL-10, IL-7A and TNF-α. (D-F) Merged figures showing the mean fluorescence intensities (MFI) of each inflammatory cytokine in the homogenates prepared from footpad lesional tissue in WT and NLRP3–/– mouse models at indicate time points (dpi). (G) Cytokine concentrations were quantified using the PE mean fluorescence intensity (MFI), which was interpolated against a standard curve generated from serially diluted cytokine standards. This analysis was performed with FCAP Array V3.0 software. As a negative control, assay diluents were processed in parallel with the samples.

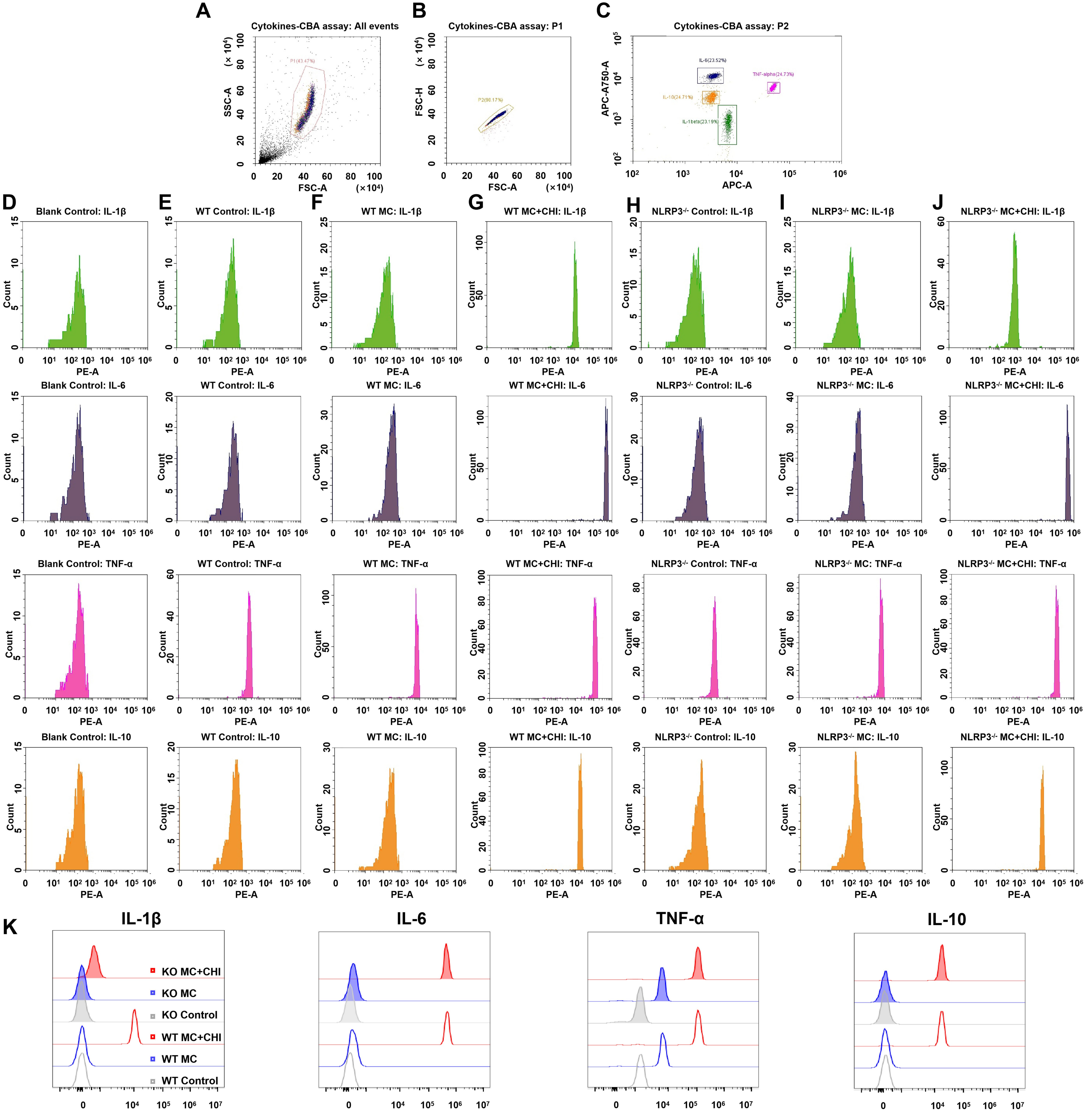
A
Cytokines-CBA assay: All events
SSC-A (× 10⁴)
FSC-A (×10⁴)
P1(43.47%)
B
Cytokines-CBA assay: P1
FSC-H (× 10⁴)
FSC-A (×10⁴)
P2(98.17%)
C
Cytokines-CBA assay: P2
APC-A750-A
APC-A
IL-6(23.52%)
TNF-alpha(24.73%)
IL-10(24.71%)
IL-1beta(23.19%)
D
Blank Control: IL-1β
E
WT Control: IL-1β
F
WT MC: IL-1β
G
WT MC+CHI: IL-1β
H
NLRP3-/- Control: IL-1β
I
NLRP3-/- MC: IL-1β
J
NLRP3-/- MC+CHI: IL-1β
Blank Control: IL-6
WT Control: IL-6
WT MC: IL-6
WT MC+CHI: IL-6
NLRP3-/- Control: IL-6
NLRP3-/- MC: IL-6
NLRP3-/- MC+CHI: IL-6
Blank Control: TNF-α
WT Control: TNF-α
WT MC: TNF-α
WT MC+CHI: TNF-α
NLRP3-/- Control: TNF-α
NLRP3-/- MC: TNF-α
NLRP3-/- MC+CHI: TNF-α
Blank Control: IL-10
WT Control: IL-10
WT MC: IL-10
WT MC+CHI: IL-10
NLRP3-/- Control: IL-10
NLRP3-/- MC: IL-10
NLRP3-/- MC+CHI: IL-10
Count
PE-A
K
IL-1β
IL-6
TNF-α
IL-10
KO MC+CHI
KO MC
KO Control
WT MC+CHI
WT MC
WT Control

Supplementary Figure 4. Representative flow cytometric plots showing the gating of CBA flex set analysis for mBMDM cytokines: (A and B) Single cells were gated as P1 (FSC-A *vs.* SSC-A) and then as P2 (FSC-A *vs*. FSC-H). Numbers indicate the percentage of parent-gated cells. (C) Capture antibodies immobilized on beads with distinct APC/APC-A750 fluorescence intensities enable multiplex detection of IL-1β, IL-6, TNF-α, and IL-10. (D-J) Levels of IL-1β, IL-6, TNF-α, and IL-10 (from top to bottom) in culture supernatants of wild-type (WT) or NLRP3-/- murine bone marrow-derived macrophages (mBMDMs). Cytokines were detected using PE-conjugated antibodies from the Flex Set. Panels show results under the following conditions: (D) blank control; (E) untreated WT mBMDMs; (F) WT mBMDMs stimulated with muriform cells; (G) WT mBMDMs stimulated with chitinase-pretreated muriform cells; (H) untreated NLRP3-/- mBMDMs; (I) NLRP3-/- mBMDMs stimulated with muriform cells; (J) NLRP3-/- mBMDMs stimulated with chitinase-pretreated muriform cells; (K) Merged figures showing the mean fluorescence intensities (MFI) of inflammatory cytokines across experimental and control groups. KO: NLRP3–/–; MC: muriform cells; CHI: chitinase; Control: untreated mBMDMs; WT: wild-type.

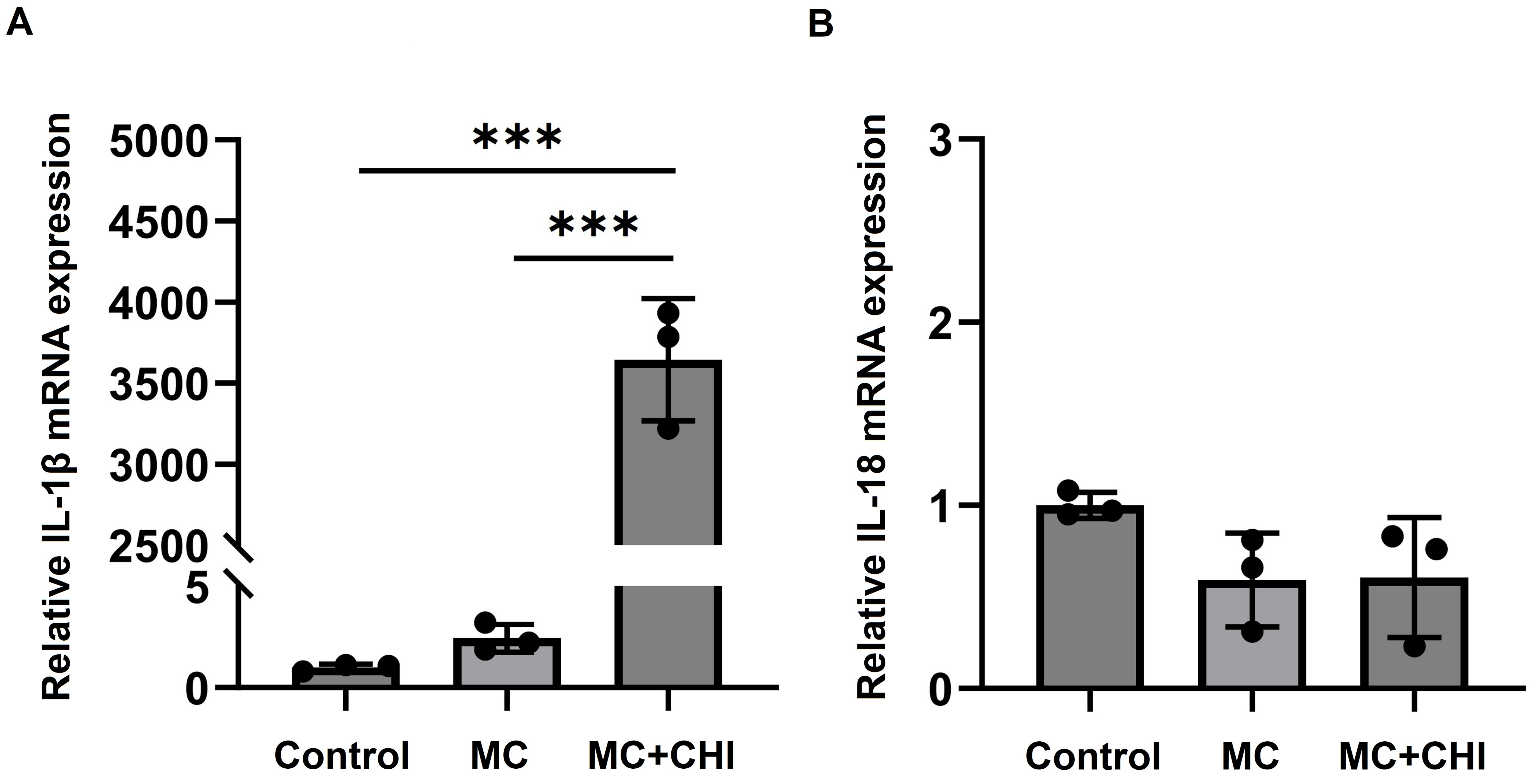
A
B
Relative IL-1β mRNA expression
5000
4500
4000
3500
3000
2500
5
0
***
***
Control
MC
MC+CHI
Relative IL-18 mRNA expression
3
2
1
0
Control
MC
MC+CHI

Supplementary Figure 5. Relative mRNA levels of IL-1β and IL-18. IL-1β (A) and IL-18 (B) mRNA levels in WT mBMDMs were measured before and after 14-h stimulation with heat-inactivated muriform cells (MOI = 3:1), with or without chitinase pretreatment. Gene expression levels were quantified using the $2^{-\Delta\Delta Ct}$ method. All RT-PCR assays were performed using an ABI Stepone Plus Real-Time PCR System (Applied Biosystems, USA). Data are presented as mean ± SD (n = 3). Compared with control or MC group, *** $p < 0.001$. Control: untreated mBMDMs; MC: mBMDMs stimulated with muriform cells; MC+CHI: mBMDMs stimulated with muriform cells with chitinase pretreatment.
